# The trafficking of bacterial type rhodopsins into the *Chlamydomonas* eyespot and flagella is IFT mediated

**DOI:** 10.1038/srep34646

**Published:** 2016-10-03

**Authors:** Mayanka Awasthi, Peeyush Ranjan, Komal Sharma, Sindhu Kandoth Veetil, Suneel Kateriya

**Affiliations:** 1 Department of Biochemistry, University of Delhi South Campus, Benito Juarez Road, New Delhi, India-110021; 2 School of Biotechnology, Jawaharlal Nehru University, New Delhi, India-110067

## Abstract

The bacterial type rhodopsins are present in all the three domains of life. In contrast to the animal type rhodopsin that performs mainly sensory functions in higher eukaryotes, the bacterial type rhodopsin could function as ion channel, pumps and as sensory proteins. The functioning of rhodopsin in higher eukaryotes requires the transport of rhodopsin from its site of synthesis to the ciliated outer segment of the photoreceptive cells. However, the trafficking of bacterial type rhodopsin from its site of synthesis to the position of action is not characterized. Here we present the first report for the existence of an IFT-interactome mediated trafficking of the bacterial type rhodopsins into eyespot and flagella of the *Chlamydomonas*. We show that there is a light-dependent, dynamic localization of rhodopsins between flagella and eyespot of *Chlamydomonas*. The involvement of IFT components in the rhodopsin trafficking was elucidated by the use of conditional IFT mutants. We found that rhodopsin can be co-immunoprecipitated with the components of IFT machinery and with other protein components required for the IFT-cargo complex formation. These findings show that light-regulated localization of rhodopsin is not restricted to animals thereby suggesting that rhodopsin trafficking is an IFT dependent ancient process.

Rhodopsin is the key photoreceptor that mediates the photobehavioural responses in different organisms^1^,^2^,^3^. The placement of rhodopsins at the proper site in the cell is crucial for optimal photoperception and the subsequent photoresponses^3^,^4^. Metazoans position rhodopsins in the ‘outer segment’ (OS) of the rod cells to detect light^5^,^6^. Lower motile organisms like the unicellular algae, *Chlamydomonas reinhardtii,* possess bacterial type rhodopsins in the eyespot for photosensing^7^. The non-motile organisms have spectrally tuned rhodopsin to match the wavelength of light abundant in their niche^8^. In mammals, rhodopsins are transported from the cell body to the OS through connecting cilium by the intraflagellar transport (IFT) machinery^9^,^10^.

IFT is a highly orchestrated and dedicated means of protein transport in the cilia/flagella^11^. The assembly, maintenance and functioning of these sensory organelles require IFT^12^,^13^. In IFT, large protein complexes called IFT trains move bi-directionally, i.e., from the ciliary base towards the tip (microtubule plus end) of the cilia (anterograde)^14^ and backwards (retrograde)^15^. Anterograde and retrograde movements are powered by the molecular motor proteins, kinesin-2^14^,^16^,^17^ and cytoplasmic dyneins^18^,^19^,^20^,^21^, respectively. These motor proteins in association with IFT particles, carry some of the ciliary cargoes but some ciliary cargoes are known to be carried independent of these motor proteins^22^,^23^. IFT particles have at least 22 subunits and are composed of sub-complexes IFTA (~6 subunits) and IFTB (~16 subunits)^24^,^25^,^26^. These sub-complexes serve as adaptors between IFT motors and the ciliary cargoes^27^,^28^,^29^,^30^,^31^,^32^. Defects in the IFT and ciliogenesis are linked with many developmental disorders and diseases collectively referred to as ciliopathies^33^,^34^,^35^. The ciliopathies related to rhodopsin trafficking lead to defects like impaired vision, irreversible blindness, Retinitis Pigmentosa (RP; OMIM: 268000), Leber Congenital Amarouses (LCA; OMIM: 204000).

*Chlamydomonas* possesses seven different bacterial type rhodopsins called chlamyopsins^36^. Chlamyopsin3 and 4 (Cop3 and Cop4) are involved in the photo-behavioral (phototaxis and photophobic) responses and because of their light-activated ion channel activities, these have been renamed as channelrhodopsin 1 (ChR1)^37^ and channelrhodopsin 2 (ChR2)^38^, respectively. Channelrhodopsins mediate photoreceptor current in the eyespot and also trigger the flagellar photocurrent that in turn brings about the change in calcium flux across the membrane^2^,^7^,^39^,^40^. Trans-membrane calcium flux initiates a cascade of electrical responses causing depolarization of the cell and ultimately controls the flagellar beating pattern^41^,^42^. Another photoreceptor protein (phototropin) has been recently observed to influence eyespot development, ChR1 regulation and phototactic behavior^43^. Studies related to the cellular localization of ChR1 showed that channelrhodopsins are localized in the eyespot of *Chlamydomonas*^44^,^45^ with the help of cytoskeletal components^46^. However, how *Chlamydomonas rhodopsins* are trafficked inside the cell and how this transport is regulated are largely unknown.

This report provides the first evidence for the involvement of intraflagellar transport (IFT) in the ferrying of bacterial type rhodopsin proteins. IFT molecular motors and IFT particles were found to be involved in the trafficking of Chlamyopsin8/Cop8 (novel rhodopsin identified in this study) and ChR1 into the flagella, in a light dependent manner. Use of different conditional IFT mutants enabled us to monitor the fate of Cop8 and ChR1 in IFT depleted yet flagellated *Chlamydomonas* cells. The interaction studies provided the evidences of the interaction between *Chlamydomonas* rhodopsins and the components of IFT machinery along with the proteins involved in the IFT-cargo complex formation. Our data leads to a model in which IFT machinery participates in the rhodopsin transport in unicellular eukaryotic green algae *Chlamydomonas reinhardtii*. It suggests that IFT mediated trafficking of rhodopsin is not only restricted to vertebrates but also occurs in lower eukaryotes.

## Results

### Light regulates the localization of bacterial type rhodopsins in the cellular compartments

**Figure 1:**
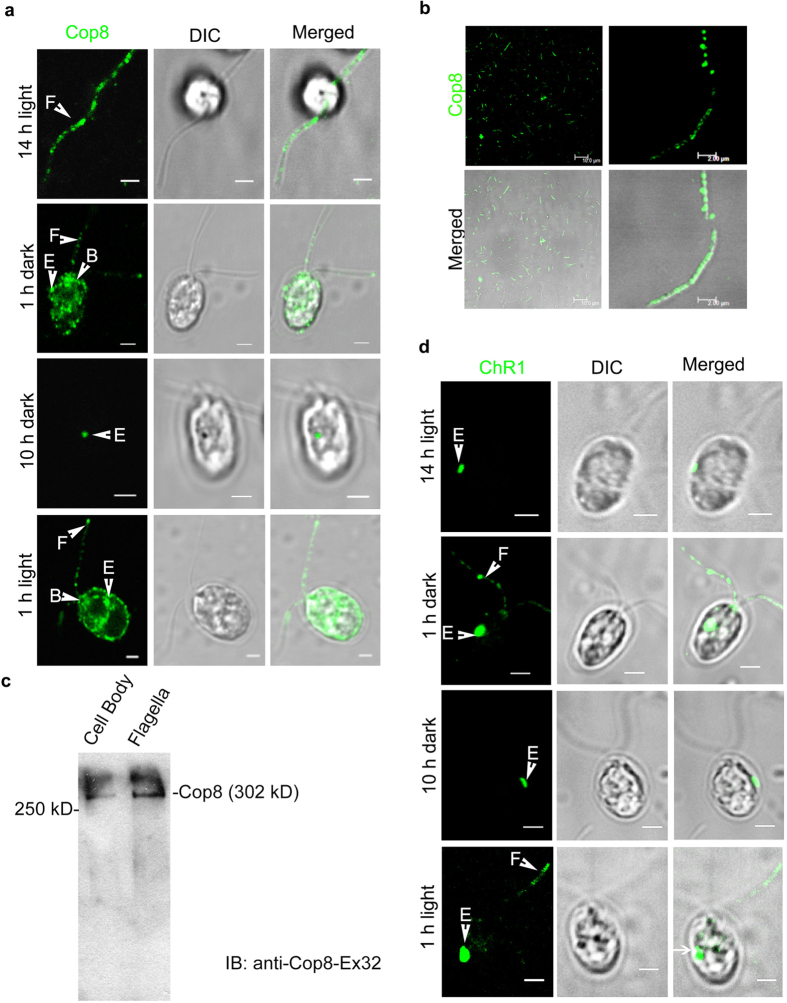
Light regulated trafficking of the Cop8 and ChR1 in flagella. (**a**) Immunostaining of Cop8 with anti-Cop8Ex32 (green) in *Chlamydomonas reinhardtii* cells at different time points of 14/10 h light-dark cycle as indicated in the left of each panel. Differential interference contrast (DIC) images are presented to show the intact cell body and flagella. Merged images are also shown to compare cellular localization pattern of a Cop8 signal within the cell. (**b**) Immunostaining of Cop8 in isolated flagellar fraction. Absence of any cell body in the DIC image showed the purity of the isolated flagellar fraction. Cop8 was nicely punctated in the flagella, as shown in the zoomed-in merged image. (**c**) Immunodetection of Cop8 in isolated cell bodies and flagellar fractions of *C.reinhardtii* (**d**) Immunostaining of ChR1 using anti ChR1-peptide antibody (green) along with DIC and merged images at different time points of the light-dark cycle. Arrows indicate the cellular localization of Cop8 and ChR1 in eyespot (E), flagella (F) and basal bodies (B). Scale bars represent 2 μm.

To decipher the mode of rhodopsin trafficking in *Chlamydomonas* the light synchronized *C. reinhardtii* cells grown under 14 h light/10 h dark cycle were utilized. Cellular localization studies of bacterial/archaeal type rhodopsin proteins Channelrhodopsin 1 (ChR1) and the newly identified rhodopsin called Chlamyopsin-8 (Cop8) were performed at different time points of 14 h light/10 h dark cycle. For conserved domain architecture of different algal rhodopsin including ChR1 and Cop8, see Supplementary Fig. 1a–i. Immunolocalization of Cop8 was observed using antibodies generated against two different regions of Cop8 protein (for Cop8 antibody details see Supplementary Fig. 2a–c). In the 14 h light adapted cells, Cop8 signal was localized in the flagella of ~80% of the cell population, ~10% of the cells showed Cop8 in the eyespot and ~10% of cells showed Cop8 signal both in the eyespot and flagella (Fig. 1a; 14 h light). Dark-onset altered the localization of Cop8 and it was found to be localized in the eyespot of ~67% of the cells, in the flagella of ~75% cells and in both eyespot and flagella of 42% of cells (Fig. 1a; 1 h dark incubation). After a complete dark cycle, Cop8 was localized mainly in the eyespot (~78%) and rarely in flagella (<20%) of the observed cell population (Fig. 1a; 10 h dark). However, on the onset of the light cycle, a reversal of Cop8 localization was observed and it was now mainly localized in the flagella in ~75% of the cells and in the eyespot of only ~20% of the cell population (Fig. 1a; 1 h light). Statistical data proved the localization patterns of Cop8 in the eyespot and flagella was significant (data analysis of ~200 cells; the number of experiments, n = 5; Supplementary Fig. 2d). Cop8 nicely decorated the isolated flagella of the *C. reinhardtii* (Fig. 1b; left and right panels). Cellular detection of Cop8 (302 kD) confirmed its presence in both cell body and flagella (Fig. 1c). However, depletion of Cop8 was observed in the flagella of the dark-adapted cells when compared with that grown in white light and in blue light (Supplementary Fig. 3).

Light dependent localization patterns of ChR1 were performed and observed using anti-ChR1 peptide antibody (for ChR1 antibody details, see Supplementary Fig. 4a–e). After a complete light cycle, ~98% cells showed ChR1 localization predominantly in the eyespot region whereas ~22% cells showed ChR1 localization in the flagella as well (Fig. 1d; 14 h light). Upon dark onset, ChR1 was seen to be present both in the flagella and eyespot of the *Chlamydomonas* cells (Fig. 1d; 1 h dark). After a complete dark cycle, ChR1 was mainly localized in the eyespot (~90% of the cells; Fig. 1d; 10 h dark). Upon light onset the localization pattern of ChR1 was observed to be similar to that of the dark onset condition and present in both eyespot and flagella (Fig. 1d; 1 h light). Statistical analyses were performed and the localization patterns of ChR1 observed during light–dark transitions were found to be significant (~200 cells; n = 5; Supplementary Fig. 4f). Protein blotting showed a reduced ChR1 expression level in light as compared to that of the dark grown cells (Supplementary Fig. 4g). These results showed that there is shuttling of the rhodopsin between cell body and flagella in a light-dependent manner. Since we had observed statistically most significant difference in the localization pattern of rhodopsins (Cop8 and ChR1) between eyespot and flagella during transitions from light to dark and vice-versa, all of the further experiments were performed in the cells grown for 10 h dark followed by 1 h light.

### Flagellar entry of Cop8 and ChR1 is restricted in anterograde IFT mutants

**Figure 2:**
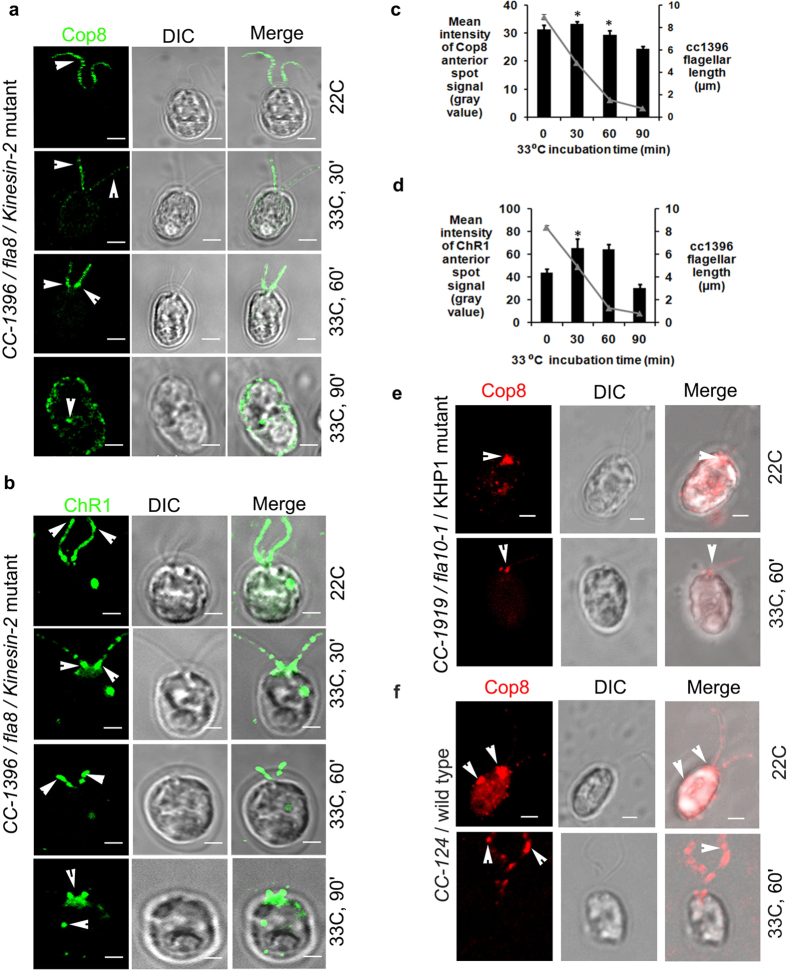
Flagellar trafficking of both Cop8 and ChR1 is affected in anterograde and retrograde IFT motor protein mutants. (**a,b**) Cellular localization of Cop8 (green) in (**a)** and ChR1 (green) in (**b**), in fla8/Kinesin-2 mutant under permissive (22°C) and non-permissive temperature (33 °C). Incubation time at non-permissive temperature is mentioned at left side of the respective panel. For each representative image, DIC and merged images are also presented. (**c,d**) Quantitation of mean fluorescence intensity of Cop8 and ChR1 of the anterior spot signal are shown in (**c** and **d)**, respectively. N = 200 cells counted per five independent experiments, for each conditions. Error bars represent standard deviation (s.d.). The significance (two-tailed t-test) is indicated, *P < 0.05, **P < 0.001. (**e,f**) Immunostaining of Cop8 in fla10/KHP1 mutant in **e** and in wild type cells in (**f)** (red) at 22 °C/dark and after 60 min of incubation at 33 °C/light. Merged images represent the accumulation of Cop8 at the anterior end, near the basal bodies. Scale bars represent 2 μm.

To decipher the mechanism of rhodopsin trafficking and the involvement of IFT machinery in rhodopsin shuttling, *Chlamydomonas* mutant strains defective in IFT motor proteins were utilized. The role of anterograde IFT motor proteins in the localization of Cop8 and ChR1 was studied in IFT motor defective strains *CC-1396/fla8/kinesin-2* mutant and *CC-1919/fla10–1/KHP1* mutant. At permissive temperature (22 °C), *fla8* mutants have phenotypes similar to wild type whereas at non-permissive temperature (33 °C) this mutant tends to reabsorb its flagella and flagellar proteins accumulate at the base of the flagella (Supplementary Fig. 5a). Two different regions of interest (ROI) of *Chlamydomonas* cells were compared (i.e. basal body and eyespot; Supplementary Fig. 5b). Under permissive temperature (22 °C), Cop8 was mostly localized in the flagella of the *fla8* mutants (Fig. 2a). The Cop8 signal in the flagella started to diminish along with flagellar reabsorption and Cop8 was eventually mislocalized in the cell body near cellular periphery and plasma membrane (Fig. 2a; 33 °C; 30–90 min). Quantitative analysis suggested sudden increase of Cop8 signal near anterior end with decrease in flagellar length, when cells were shifted from permissive to non-permissive temperature conditions (Fig. 2c).

At permissive temperature ChR1 was localized in the flagella and the eyespot of *fla8* (Fig. 2b; 22 °C) and accumulated near the basal body at non-permissive temperature (Fig. 2b; 33 °C, 30–90 min). Like that of Cop8, incubation of *fla8* cells at 33 °C for 30 min resulted in a sudden increase in the mean signal intensity of ChR1 at the anterior spot region, which decayed later with an increase in the incubation time (n = 3; Fig. 2d). Another mutant *fla10-1*, which exhibits a temperature sensitive point mutation in the gene for kinesin homologous protein (KHP1) and at non-permissive temperatures makes the flagellar precursors unable to enter the flagella^14^. Cop8 and ChR1 were observed in the dark adopted *fla10-1* cells growing at permissive temperature and were looked for the changes in the localization pattern when shifted to non-permissive temperature, where IFT was supposedly abolished. The *fla10-1* cells growing at 22 °C in dark when transferred to 33 °C in light for 60 min, showed accumulation of Cop8 near the basal bodies in both fully absorbed or the cells with shortened flagella (Fig. 2e, see also Supplementary Fig. 5c). ChR1, in addition to its presence in the eyespot was also accumulated near the basal bodies (Supplementary Fig. 5d). The presence of ChR1 in the eyespot diminished significantly in a time-dependent manner at 33 °C in both *fla8* and *fla10-1* mutants (Supplementary Fig. 5e and f, respectively). However, in *Chlamydomonas* wild type cells *CC-124,* the distribution of Cop8 in the flagella was found unaffected even at non-permissive temperature. When *CC-124* cells completing 10 h of dark cycle at 22 °C were incubated for 1 h at 33 °C in light, Cop8 decorated the flagella of *Chlamydomonas* cell (Fig. 2f).

### Cytoplasmic dynein motors play active role in the trafficking of Cop8 and ChR1

**Figure 3:**
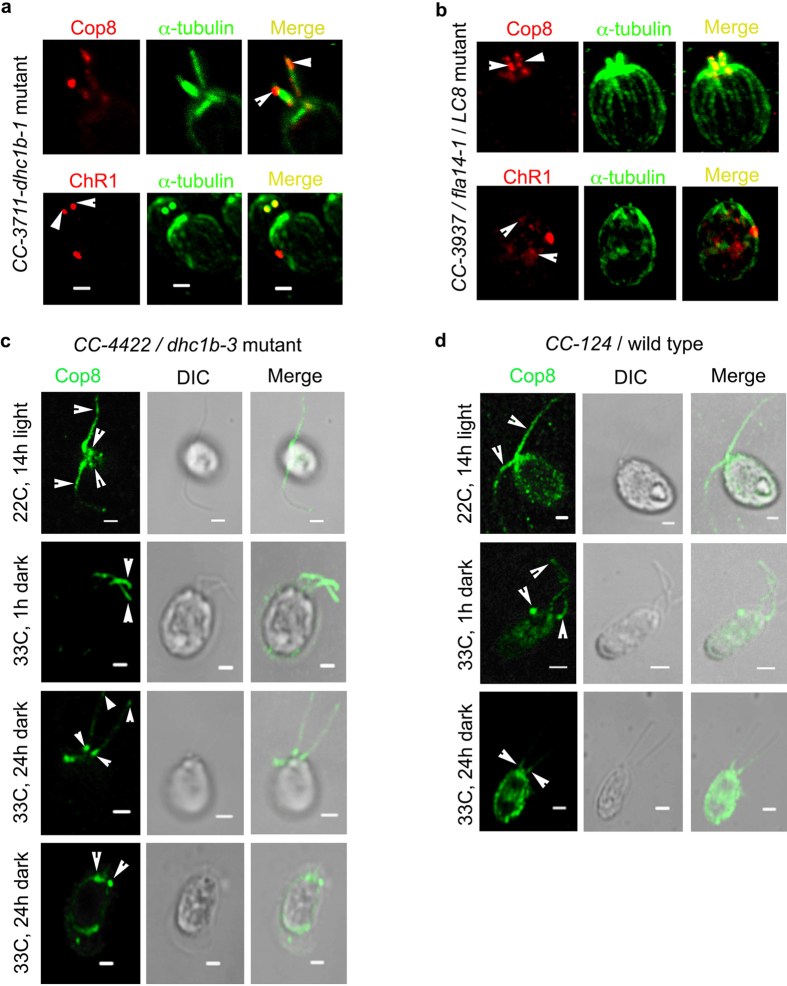
Trafficking of Cop8 and ChR1 is affected in retrograde IFT motor protein mutants. (**a**) Cellular localization of Cop8 and ChR1 in dhc1b-1 mutant. Red represents fluorescence signal for Cop8/ChR1 and green as α-tubulin. Both the proteins were accumulated at the tip of stumped flagella. Merged images represent the accumulation of Cop8 and ChR1 at the tip of stumped flagella. (**b**) Immunostaining of Cop8 and ChR1 (red) in cc3937/LC8 mutant along with α-tubulin (green). Merged images represents the accumulation of Cop8 near the basal bodies and mislocalization of ChR1in the cell body. (**c**) Fate of Cop8 localization in dhc1b-3 mutant at permissive and non-permissive temperature. Cells grown at 22 °C in light showed flagellar localization of Cop8. When these cells are transferred at 33 °C in dark for 1 h, Cop8 failed to come out of flagella and even after 24 h of dark incubation, in flagellated cells, Cop8 was accumulated at the flagellar tip and near basal bodies. In the cells with reabsorbed flagella, Cop8 was accumulated at the basal bodies. (**d**) Cop8 localization at 22 °C (light) and 33 °C (dark) in wildtype cells. Incubation time at non-permissive temperature is mentioned at left side of the respective panel. For each representative image, DIC and merged images are also presented. Scale bars represent 2 μm.

Localization of Cop8 and ChR1 was delineated in the retrograde IFT mutant strains *CC-3711*/ *dhc1b-1, CC-3937*/ *fla14-1* and in temperature sensitive mutant *CC-4422/ dhc1b-3*. In *dhc1b-1* mutant, the anterograde IFT machinery is intact and thus the anterograde transport of flagellar proteins is normal but it has a defective retrograde IFT. Cop8 and ChR1 were accumulated at the stumped flagella of *dhc1b-1* mutant (Fig. 3a; upper and lower panels, respectively). In retrograde IFT mutant *CC-3937/fla14-1* that show impaired motility due to defect in 8 kD dynein light chain *LC8*, Cop8 localization was mainly found in the basal body area, and peribasal region whereas ChR1 was mislocalized in the cell body (Fig. 3b; upper and lower panels).

To monitor the localization pattern of rhodopsin in IFT defected yet flagellated *Chlamydomonas* cells, *CC-4422/ dhc1b-3* mutant was utilized^47^. These mutants despite of the depletion of IFT machinery at 33 °C could possess near normal flagella. Cop8 decorated the flagella of *dhc1b-3* mutant when cells were grown at 22 °C, (Fig. 3c, 22 °C, 14 h light) whereas after incubation for 1 h at 33 °C in dark, Cop8 was accumulated towards the proximal ends of the flagella (Fig. 3c, 33 °C, 1 h dark). After 24 h of incubation at 33 °C in dark, some of the *dhc1b-3* cells still had flagella whereas some had fully absorbed flagella. At this time point, in the flagellated *dhc1b-3* cells, Cop8 was present rarely in the flagella but mainly in the basal bodies, whereas in the cells with fully absorbed flagella Cop8 was found accumulated at the basal bodies. In the wild type *Chlamydomonas* cells *CC-124,* the localization pattern of Cop8 in flagella and eyespot wasn’t found much affected due to temperature shift from 22 °C (Fig. 3d, 22 °C) to 33 °C (Fig. 3d, 33 °C, 1 h). Cop8 was distributed throughout the flagellar length. However, after prolonged incubation at 33 °C for 24 h at dark, it was observed to be mislocalized in the cell body (Fig. 3d, 33 °C, 24 h).

Photobehavioral responses of *Chlamydomonas* are mediated by the rhodopsins^47^. Our immunolocalization results indicated the reduction in the localization of Cop8 in the flagella of *dhc1b-3* mutant, when cells under light cycle at 22 °C, were shifted to the dark at 33 °C. Earlier, the phototaxis assay of *dhc1b-3* cells had been shown to undergo a switch from negative to positive phototaxis when shifted from permissive 21 °C to non-permissive temperature 34 °C conditions^47^. We carried out phototaxis assay, in-order to find out whether alteration in the light dependent rhodopsin localization in *dhc1b-3* mutant could also affect the phototaxis ability of these cells,. In our phototaxis assay the *Chlamydomonas* wild type *CC-124* and mutant *CC-4422/dhc1b-3* strains under light cycle at 22 °C were compared with the cells incubated for 7 h in dark at 33 °C. Wild type *C. reinhardtii* cells showed phototaxis upon illumination both in the cells growing at 22 °C and after incubation at 33 °C for 7 h in dark, whereas, phototaxis ability was greatly lost in *dhc1b-3* mutant under similar transition (Supplementary Fig. 5g). These findings provided the strong evidences for the role of retrograde IFT motor proteins in the Cop8 and ChR1 trafficking.

### IFT particles mediate flagellar transport of the Cop8 and ChR1

**Figure 4:**
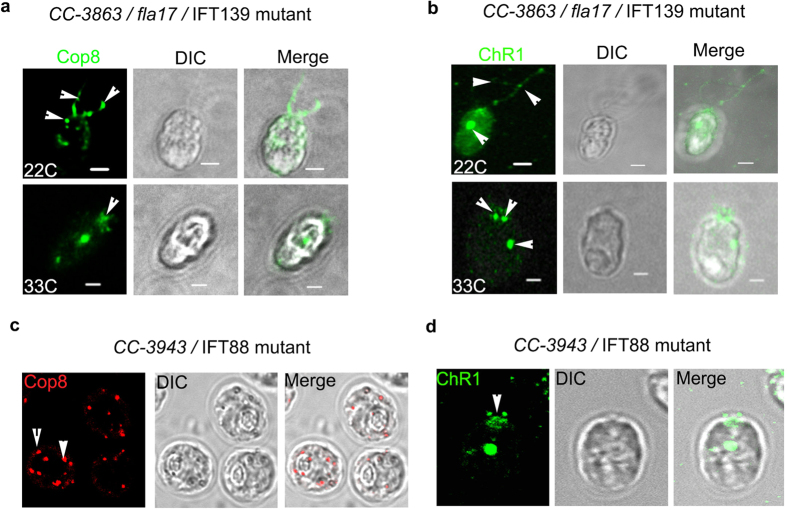
Cellular trafficking of Cop8 and ChR1 is altered in *Chlamydomonas* IFT particle mutants. (**a,b**) Immunostaining of Cop8 (**a**) and ChR1 (**b**) (green) in *CC-3863*/IFT139 mutant, under permissive (22 °C, upper panel) and non-permissive temperature (33 °C, lower panel). Arrow indicates the mislocalization of Cop8 and ChR1 in IFT139 mutant. (**c,d**) Cellular localization of Cop8 (**c**, red) and ChR1 (**d**, green) in *CC-3943*/IFT88 mutant in cells grown at 25 °C. Mislocalization of Cop8 in the cell body and accumulation of ChR1 near the basal body of IFT88 mutant were indicated by arrowheads. DIC and merged images are also presented. Scale bars represent 2 μm.

To elucidate the role of IFT particle subunit in the flagellar trafficking of Cop8 and ChR1, we used *CC-3863/fla17 Chlamydomonas* mutant strain, which is defective in IFT139 (IFTA subunit) and shows temperature dependent IFTA defect. We reasoned that, if IFTA and IFTB sub-complexes are involved in the rhodopsin localization, then the localization pattern of Cop8 and ChR1 must be affected in the *Chlamydomonas* mutants’ defective in one or other IFT particle subunits. The *fla17* cells has temperature dependent IFT defect, hence the cellular localization of Cop8 and ChR1 were studied at both 22 °C and 33 °C in this mutant. At 22 °C when these mutants undergo normal IFT, Cop8 and ChR1 were observed in the flagella but in comparatively less amount than that of the wild type cells. The reason for this might need further investigation in this direction. However, at 33 °C, flagella were reabsorbed and both Cop8 and ChR1 were mostly accumulated at the anterior end near the basal bodies (Fig. 4a,b; upper and lower panels). Fate of Cop8 and ChR1 was also observed in another IFT particle subunit mutant strain *CC-3943,* which is defective in IFT88 (IFTB subunit). IFT88 is essential for the assembly and maintenance of the outer segment of vertebrate photoreceptor cells^48^. In contrast to wild type cells, *Chlamydomonas* IFT88 mutant do not possess flagella hence motility is impaired in these mutants. IFT88 mutants showed mislocalization of Cop8 (Fig. 4c) and ChR1 (Fig. 4d) in the cell body and accumulation of ChR1 near the anterior end (Fig. 4d). Cellular localization of Cop8 in *CC-477/ bld1-1* cells, which is IFT52 mutant and do not possess flagella showed the accumulation of Cop8 in the basal bodies (Supplementary Fig. 5h). These findings elucidate the participation of IFT particles in the trafficking of rhodopsin in *Chlamydomonas*.

### Proteomics evidences of the involvement of IFT components in the trafficking of ChR1

**Figure 5:**
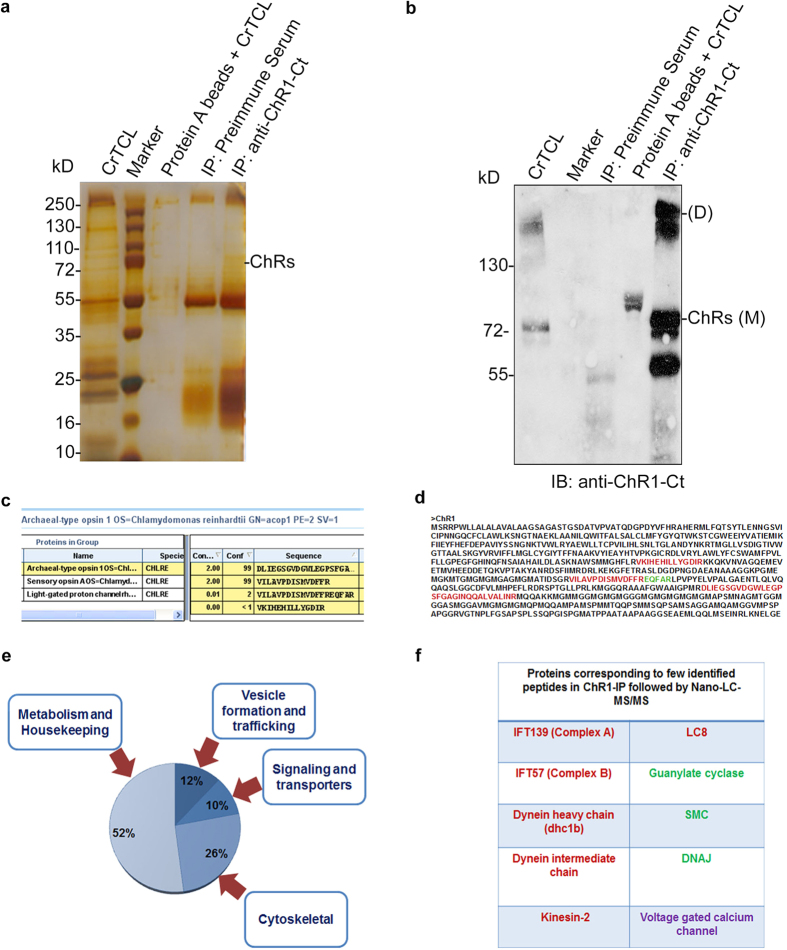
ChR1 co-immunoprecipitates IFT protein components from *C. reinhardtii* total cell lysate (CrTCL). (**a**) Proteins co-immunoprecipitated using anti-ChR1-Ct as observed by silver staining of the protein bands resolved on 12% SDS-PAGE. (**b**) Immunoblotting of ChR1 using anti-ChR1-Ct in the ChR1-IP elution fractions resolved in 10% SDS-PAGE. (**c**) ChR1 peptides identified in ChR1 proteomics by Protein Pilot software. (**d**) Identified peptides of ChR1 protein sequence marked in a full-length ChR1 protein sequence. Red region depicts the peptides belonging to both ChR1 and ChR2 proteins whereas green region shows peptide unique to ChR1 protein. (**e**) Pi-diagram representing the percentage of different categories of proteins co-immunoprecipitated in ChR1-IP. Categories of co-eluted proteins include cytoskeletal proteins, signaling and transporter proteins, vesicle formation or trafficking proteins and metabolism or housekeeping proteins. (**f**) List of proteins corresponding to the few identified peptides in ChR1-IP followed by Nano-LC-MS/MS. Red labeled proteins are known to be involved in intraflagellar transport. Green labeled proteins are involved in IFT-cargo complex formation. Putative voltage-gated calcium channel (VGCC) involved in the generation of photocurrent in *Chlamydomonas* is labeled in purple.

To further explore out the association between bacterial rhodopsins and IFT proteins, the proteomics experiment was performed, where ChR1 was immunoprecipitated from *C.reinhardtii* Total Cell Lysate (CrTCL) using anti-ChR1 (Fig. 5a) and the co-immunoprecipitated proteins were identified by Nano-LC-MS/MS. The presence of ChR1 in the IP eluent was confirmed by immunoblotting with anti-ChR1-Ct (Fig. 5b). Identification of the other putative interactors of ChR1 was made on the basis of their presence in ChR1-IP but not in the negative control where pre-immune serum was used. ChR1 is a seven transmembrane (TM) protein with extended C-terminal region. Three of the four identified peptides of ChR1 were from the C-terminal region and other from the TM region (Fig. 5c,d). Identified proteins were found to be components of different cellular functions such as trafficking, signaling, metabolism and housekeeping functions (Fig. 5e). Interestingly, the components of the IFT machinery Dhc1b, LC8, Kinesin-2, IFT139, IFT52 and several flagellar associated proteins (FAPs) were also detected (Fig. 5f). Although only single peptide of these proteins were identified in ChR1 proteomics, we decided to substantiate the role of IFT component in ChR1 trafficking by identifying the interaction between ChR1 and few of the identified trafficking proteins.

**Figure 6:**
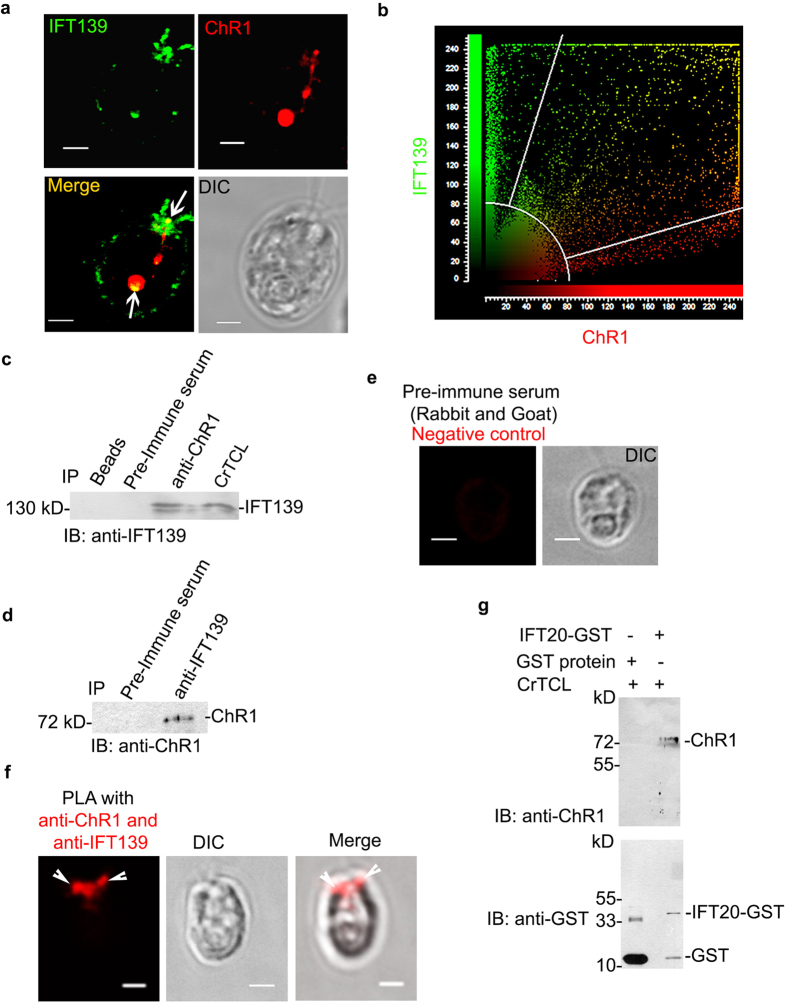
ChR1 and Cop8 associate with IFT particles in *C. reinhardtii* (**a**) Colocalization of IFT139 and ChR1 mainly in eyespot and near basal bodies in wild-type *CC-124* cells. Confocal micrographs of the *C. reinhardtii* cell labeled with IFT139 (green) and ChR1 (red). The merged image is shown in which yellow region represents colocalization. DIC image is also shown. (**b)** Scatter plot of co-localization signal of ChR1 with IFT139. Maximum overlapping red (ChR1) and green (IFT139) pixels at diagonal indicates significant co-localization between two pixels. **(c,d**) Immunoprecipitation (IP) of ChR1 in **c** and IFT139 in (**d)** using anti-ChR1-peptide and anti-IFT139 antibodies, respectively followed by immunoblotting (IB) with indicated antibodies. Protein bands of 139 kD in (**c)** and 78 kD in (**d)** represents IFT139 and ChR1, respectively. IP with pre-immune serum served as negative control in both the cases. (**e**) Proximity ligation assay (PLA) using the pre-immune serum (negative control). (**f**) PLA using anti-ChR1 and anti-IFT139 together with DIC and merged images. (**g**) GST pull-down of ChR1 from *C. reinhardtii* total cell lysate (CrTCL) using IFT20-GST as bait. Immunoblotting with anti-ChR1 (upper panel) peptide indicated that ChR1 was pulled down with IFT20-GST protein but not with the recombinant GST protein. Immunoblotting of the same membrane was performed with anti-GST (lower panel). Protein bands approximate to 23 kD, and 42 kD represents recombinant GST and IFT20-GST proteins, respectively. Scale bars represent 2 μm.

Antibodies against IFT139 peptide region were generated, which were checked for specificity by immunoblotting (Supplementary Fig. 6a–c). The colocalization of ChR1 and IFT139 mainly near the basal bodies of *C. reinhardtii* suggested the possibility of interaction between these proteins (Fig. 6a,b). IFT139 was identified in the ChR1-IP fraction (Fig. 6c) and ChR1 was identified in IFT139-IP (Fig. 6d). In order to further confirm the interaction in the cell, we performed the *insitu* Proximity Ligation Assay (PLA) between IFT139 and ChR1. The appearance of the red fluorescence spot near the basal bodies, specifically with ChR1 and IFT139 antibodies but not with pre-immune serum indicated interaction of IFT139 with ChR1 (Fig. 6e,f) in *Chlamydomonas* cells. It has been established that IFT20 is required for the trafficking of animal type rhodopsin in mammals^9^. We therefore tested the interaction of IFT20 with ChR1 in the CrTCL by a GST-pulldown assay using recombinant IFT20-GST. Recombinant IFT20-GST was able to pull down cellular ChR1 (Fig. 6g) thereby suggesting the interaction of IFT20 with ChR1.

### IFT cargo complex components assists the cellular trafficking of ChR1 in *Chlamydomonas*

**Figure 7:**
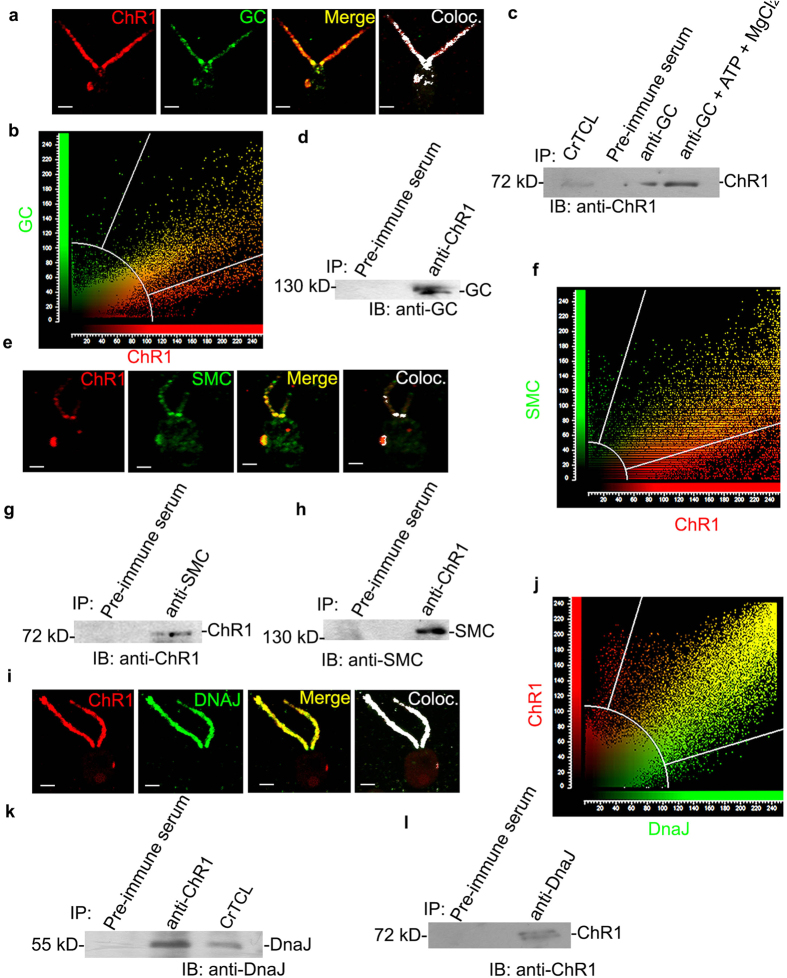
ChR1 interacts with mammalian like IFT-cargo complex components in *C. reinhardtii* (**a**) Colocalization of ChR1 (red) and Guanylate Cyclase (GC; green) in *CC-124 C. reinhardtii* cell. In the merged image, the yellow region represents colocalization. Colocalized pixels with overlap coefficient of 0.87, are depicted in the colocalized (coloc; white) image. (**b**) Scatter plot of co-localization signal of ChR1 with GC. Maximum overlapping red (ChR1) and green (GC) pixels at diagonal indicates significant co-localization between two pixels. (**c**,**d**) GC- immunoprecipitation (IP) in presence and absence of Mg^2+^ /ATP using anti-GC, in (**c)** and ChR1 with anti-ChR1-peptide in (**d)**, respectively, followed by immunoblotting with indicated antibodies. Protein bands corresponding to 78 kD in **(c)** and 130 kD in (**d)** represents ChR1 and GC respectively. (**e**) Colocalization of ChR1 (red) and SMC (green) in *C. reinhardtii* cell. The yellow region shows colocalization in the merged image. Colocalized pixels (white) with overlap coefficient of 0.81 depicted in the colocalized image. (**f**) Scatter plot of co-localization signal of ChR1 with SMC depicting maximum overlapping pixels (yellow) at diagonal. (**g,h**) IP of SMC and ChR1 using anti-SMC and anti-ChR1 peptide in (**g** and **h)**, respectively, followed by IB with indicated antibodies. Protein bands corresponding to 78 kD in (**g)** and 150 kD in (**h)** represents ChR1 and SMC, respectively. (**i**) Colocalization of ChR1 (red) and DnaJ (green) in *C. reinhardtii* cell. In the merged image, the yellow region represents colocalization. Colocalized pixels (overlap coefficient of 0.91) are depicted in the colocalized image (coloc.; white). (**j)** Scatter plot of co-localization signal of ChR1 with DnaJ depicting maximum overlapping pixels (yellow) at diagonal. **(k,l**) IP of DnaJ with anti-DnaJ in (**k**) and IP of ChR1 with anti-ChR1-peptide antibodies in (**l**) followed by IB with indicated antibodies. Protein bands corresponding to 78 kD in (**k**) and 61 kD in (**l**) represents ChR1 and DnaJ, respectively. IP with pre-immune serum served as negative control in each case. Scale bars represent 2 μm.

IFT mediated rhodopsin transport in mammals also involves the role of other proteins like Guanylate cyclase (GC), molecular chaperone protein (DnaJ) during IFT-cargo/rhodopsin complex formation. Furthermore, the structural maintenance of chromosome protein (SMC) is recently characterized for their role in rhodopsin trafficking in vertebrates. Single peptides of these proteins were also detected by the nano-LCMS/MS of the ChR1-immunoprecipitate. Antibodies against the peptide regions of GC, SMC and DnaJ were generated (For details about the sequences refer Supplementary Table 2 and for antibodies see Supplementary Fig. 6). GC and ChR1 were colocalized in the eyespot and flagella of *C. reinhardtii* cells (Fig. 7a,b). These proteins co-immunoprecipitated each other and ChR1 detected by immunoblotting showed a significant increase in band intensity when co-immunoprecipitated with GC in the presence of Mg^2+^ -ATP (Fig. 7c,d). The red fluorescence obtained in the PLA using anti-GC and anti-ChR1 (Supplementary Fig. 6h) demonstrated the interaction between ChR1 and GC *insitu*. ChR1 and SMC co-localized mainly near the basal bodies and eyespot (Fig. 7e,f). Interaction between these two proteins was further substantiated by co-IP (Fig. 7g,h). Chaperone DnaJ was also co-localized with ChR1 in the flagella of *C. reinhardtii* (Fig. 7I,j). Interaction between DnaJ and ChR1 was further confirmed by the reciprocal co-IP (Fig. 7k,l). Further, the presence of IFT139 in the GC, SMC and DnaJ co-immunoprecipitate, which contain ChR1 (Supplementary Fig. 7a), suggested the close association of these protein candidates in IFT mediated ChR1 trafficking. To confirm whether the identified interactions are ChR1 specific we checked out the interaction of LC8 and DnaJ with another photoreceptor protein channelrhodopsin2 (ChR2) of *Chlamydomonas*, (For ChR2-peptide antibody specificity see Supplementary Fig. 4b). Interestingly, ChR2 was not found to interact both with LC8 and DnaJ (Supplementary Fig. 7b–d). Observed findings well supported the possibility of interaction between ChR1 and IFT proteins. These results suggested that the involvement of IFT protein components in rhodopsin trafficking might be an evolutionarily conserved phenomenon.

## Discussion

*Chlamydomonas reinhardtii* was known to possess at least seven rhodopsin proteins called chlamyopsins (Cop) numbered from one to seven, hence designated as Cop1 to Cop7^36^. This study leads to the identification of a novel rhodopsin present in *Chlamydomonas reinhardtii*, which we named as Chlamyopsin 8 or Cop8. Cop8 is a multidomain rhodopsin where N-terminal of rhodopsin is flanked with potassium channel and cyclic nucleotide-monophosphate binding domain (cNMP), and C-terminal harbor other modular domains like, Histidine kinase (Hk) and response regulator and cyclase domains (Supplementary Fig. 1e). Immunolocalization studies of Cop8 using two different antibodies specific to this protein showed that Cop8 is present in the eyespot and flagella of *Chlamydomonas* and that its distribution was light dependent (Fig. 1a). The immunolocalization studies of ChR1 (Cop3) protein also revealed the light dependent distribution of this protein between the eyespot and flagella (Fig. 1d). Our results showed that Cop8 and ChR1 aren’t always lodged into the flagella of the *Chlamydomonas,* but their presence in the flagella depends upon the environmental light condition. The shuttling of these rhodopsins (Cop8 and ChR1) into the flagella envisaged the involvement of intraflagellar transport (IFT) machinery in transport from the site of their synthesis to the site of action.

The cellular localization of Cop8 and ChR1 in the temperature sensitive anterograde IFT mutants (*CC-1396/fla8/kinesin-2*^49^ and *CC-1919/fla10-1/KHP1*^50^) showed accumulation of Cop8 and ChR1 near basal bodies (Fig. 2). Though reduced, but the signal of Cop8 and ChR1 was also noticed in the reabsorbing flagella of *fla8* mutants at 33 °C, the temperature condition where IFT is supposedly ceased in these mutants. The presence of rhodopsins into the shortened flagella of fla8 mutants could not vouch for the clear picture of the involvement of IFT in trafficking of Cop8 and ChR1 in *Chlamydomonas*. Both Cop8 and ChR1 were nicely entrapped in the peri-basal region of *fla10-1/KHP1* mutants at 33 °C which served as an evidence for active involvement of anterograde IFT motors for the flagellar entry of these bacterial type rhodopsin proteins (Fig. 2 and Supplementary Fig. 5c,d). Quantitation of the eyespot localization signal of the Cop8 (33 °C) and ChR1 (33 °C) showed the involvement of anterograde IFT even in the eyespot localization of these proteins in *Chlamydomonas* (Supplementary Fig. 5e,f).

In retrograde motor protein mutants like *CC-3711/dhc1b-1*^51^ and *CC-3737/LC8,* both Cop8 and ChR1 failed to exit the flagellar compartment and were accumulated at tip of stumped flagella. In the flagellated *CC-4422/dhc1b-3* cells, the retrograde transport of Cop8 was abrogated at 33 °C. The eyespot localization of Cop8 and ChR1 was also affected in these mutants (Fig. 3). Furthermore, the loss of phototaxis ability of the *dhc1b-3* mutant at 33 °C clearly combines in support of the evidences for involvement of retrograde IFT motar mutants in Cop8 and ChR1 trafficking (Supplementary Fig. 5g).

IFT particle (IFTA and IFTB) and accessory proteins play major role to accomplish the process of the movement of the animal type rhodopsin in the vertebrates^9^,^10^,^52^. Earlier studies states the involvement of IFT20 in the post-Golgi trafficking and IFT88, IFT57, IFT52 in the vertebrate photoreceptor assembly and maintenance^48^,^53^,^54^. Mislocalization of Cop8 and ChR1 in the IFT139 mutant strain^55^ and in IFT88 mutant^56^ (Fig. 4) clearly supported the involvement of IFT particle in Cop8 and ChR1 shuttling between flagella and eyespot. ChR1-IP followed by nano-LC-MS/MS identified few IFT components co-immunoprecipitated along with ChR1 (Fig. 5). Among the co-immunoprecipitated proteins, IFT139, IFT20, GC, DnaJ and SMC were further studied in detail to establish their interaction with ChR1. Co-localization, PLA, Co-IP together with GST pull-down experiment provided evidence for the association of IFT particles and other IFT cargo complex components with ChR1 (Figs 6 and 7).

In the vertebrate photoreceptors, transport of rhodopsins occurs in the form of cargo-vesicles, continuously from the endoplasmic reticulum (ER) and trans-Golgi network (TGN) toward the outer segment^57^,^58^,^59^. Interaction between proteins of the transport machinery and vesicular proteins is required for such polarized transport. IFT-cargo complexes including guanylate cyclase GC1, DnaJ, rhodopsin, etc., are transported by IFT machinery upto the flagellar tip^10^. Recently, SMC proteins were found to be involved in regulation of the protein transport in primary cilia of vertebrate photoreceptors^60^. SMC was found to localize in sensory cilia, and these were proposed to play a role in microtubule dynamics and trafficking of the rhodopsins^61^. The influence of Mg^2+^ -ATP in Co-IP of ChR1 with GC suggested the importance of ATPase cycle in the formation or stabilization of GC mediated ChR1-IFT complex (Fig. 7a–d). The interaction of SMC with ChR1 indicated the role of SMC in regulating ChR1-IFT complex transport assemblies through microtubules (Fig. 7e–h). The evidences of interaction between ChR1 and DnaJ (Fig 7i–l), suggested the involvement of chaperones in the IFT regulated trafficking of rhodopsins. However, the functional significance of interaction of GC, SMC and DnaJ with ChR1 is beyond the scope of this study.

**Figure 8:**
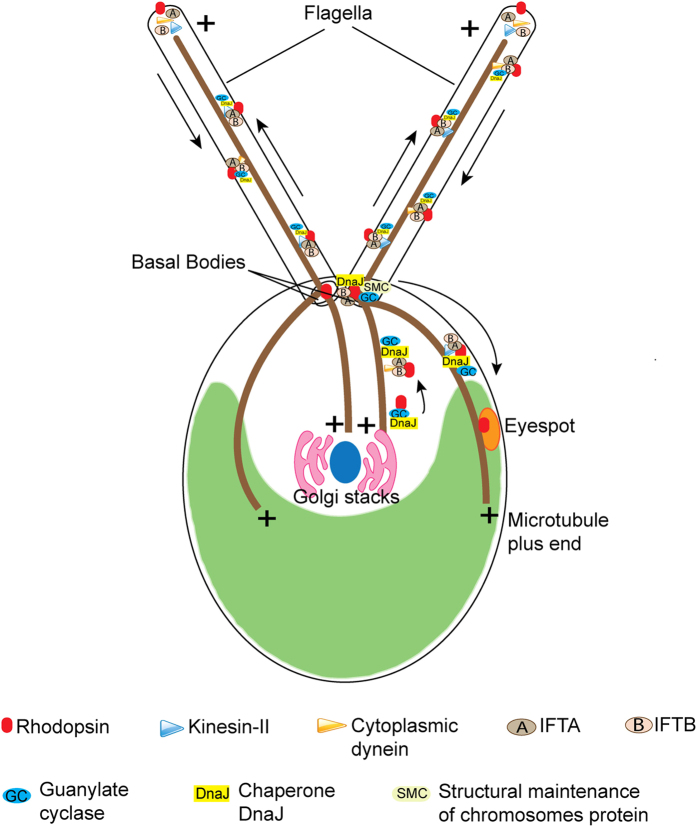
Model for the involvement of IFT-cargo complex components in the rhodopsin trafficking in *C. reinhardtii*. Flagellar transport of photoreceptor proteins in *C. reinhardtii* eyespot and flagellum involves the IFT-cargo complex components *vis* GC, DnaJ and SMC. Photoreceptors in anterograde direction would be carried by different components of anterograde IFT machinery like IFT particles, kinesin-2 and KHP1 and the turnover products are carried in retrograde direction with the help of IFT particles and cytoplasmic dynein motor proteins.

Based on experimental evidences, a working model regarding the involvement of IFT components in the flagellar transport of photoreceptors is presented (Fig. 8). We propose that the molecular components and hence the machinery involved in the trafficking of bacterial type rhodopsins (Cop8 and ChR1) in *Chlamydomonas* flagella and/or eyespot is homologous to the mammalian rhodopsins trafficking in the outer segment of the photoreceptor cells, which primarily uses IFT machinery. In future studies it will be interesting to determine the role of rhodopsins in the flagella.

The eyespot plays an accessory role in photobehavioral responses and eyeless mutant would be able to perceive and respond to light^62^. Recent study using the reactive oxygen species (ROS), leads to the identification of novel eyeless mutant of *Chlamydomonas* exhibiting strong phototaxis responses^63^. These reports support the earlier made hypothesis, that the photoperception in *Chlamydomonas* is not confined to eyespot. One possibility is that the bacterial rhodopsins localized in the flagella of *Chlamydomonas* might be responsible for a non-directional phototransduction of this organism. The eyespot-guided phototaxis is very important for the zooplankton larvae of marine invertebrates and is proposed to mediate larval swimming towards the light^64^. Recently, it has been proposed that eyespot localized opsin of the Platynereis are the photoreceptors for controlling phototaxis of this organism^61^. It would be interesting to elucidate the role of intraflagellar transport (IFT) machinery in the trafficking of opsin in the eyespot of the Platynereis, which would shed light on evolutionary link of the of the IFT mediated trafficking of the rhodopsin(s) in nature. The involvement of IFT in the eyespot localization of rhodopsin would further support its role in intracellular trafficking of proteins similar to the case of immune synapse assembly in higher animals^65^.

It is also expected that IFT-mediated trafficking of the bacterial type rhodopsin might also be operating in other flagellated protists and motile fungal zoospores in the nature. It would be interesting to elucidate the functional role of the flagella localized rhodopsin(s) in the photo-behavioral responses of this organism.

## Methods

### Bioinformatic analysis

Extensive genome database search for the archaeal rhodopsins and modular rhodopsins in eukaryotes were performed on JGI genome database of different algae (Supplementary Table 1). Multiple sequence alignment was performed using ClustalW platform under Bioedit tool (http://www.mbio.ncsu.edu/bioedit/bioedit.html). The rhodopsin domains of novel archaeal rhodopsins were identified by using conserved domain architecture retrieval tool, conserved domain database program and confirmed by sequence alignment with canonical rhodopsins.

### *Chlamydomonas* strains and media

All *Chlamydomonas* strains were obtained from the *Chlamydomonas* resource center (University of Minnesota). The *CC* strain number refers to the stock numbers from the *Chlamydomonas* culture collection in the University of Minnesota. All the strains were cultured and maintained in incubator shaker by growing it in Tris-acetate phosphate (TAP) medium under 14 h light and 10 h dark cycles at 25°C. For temperature sensitive IFT mutants permissive and non-permissive temperature for the growth were maintained at 22 °C and 33 °C, respectively.

### Antibodies

The peptide antibodies were made with the 10–15 amino acid sequence selected from target protein sequences. The antigenic properties of peptide region were determined with web-based immune epitope database prediction program (http://www.iedb.org/). The peptide was synthesized, and KLH (Keyhole Limpet Hemocyanin) was conjugated at C-terminus. Primary antibodies were generated in rabbit, mice or goat, utilizing commercial facility (Merck-Bangalore Genei, India). The organisms in which antibodies were generated are listed in Supplementary Table 2. All of the generated polyclonal antibodies were confirmed for its specificity by immunoblotting of the respective recombinant proteins (truncated or full length).

### Immunoblotting

The *Chlamydomonas* cells resuspended in preferred buffer supplemented with protease inhibitor cocktail were lysed by sonication (40% amplitude, 20 pulses of 10 seconds on/off cycles). The total cell lysate (CrTCL) mixed in equal volume with 2X Laemmli buffer was resolved on 7 to 12% SDS-PAGE and transferred to nitrocellulose membrane. The membrane was blocked with 5% fat-free milk (Titan Biotech Ltd., India) prepared in PBS (phosphate buffer saline) and supplemented with 0.1% tween-20 (Sigma, USA) at room temperature for 1 h. The blocked membrane was treated with primary antibody followed by HRP (horseradish peroxidase) conjugated secondary antibody (Sigma, USA). Immunoblotted bands were captured on Kodak film by using enhanced chemiluminescence reagents (Sigma, USA).

### Immunoprecipitation (IP) assays and mass spectrometry

*Chlamydomonas* culture was harvested by centrifugation. Cells were homogenized using cold IP-lysis buffer (1X PBS, 1% NP-40, 0.5% sodium deoxycholate, 0.1% SDS and cocktail of protease inhibitor, Sigma-Aldrich cat. no. P9599). Samples were then sonicated on ice and cell debris was clarified from the lysate by centrifugation at 13,300 rpm at 4 °C for 10 min. The lysate was pre-cleared by incubating it with a pre-immune serum of the host species in which antibody was generated along with protein A/G bead slurry at 4 °C for 1 h on a rocking platform. The bead pellet was separated by centrifugation at 13,300 rpm at 4 °C for 10 min and supernatant was used for IP. Approximately 1 μg of antibody and pre-immune serum (negative control) were mixed separately with 1 ml of pre-cleared lysate and incubated overnight at 4 °C on a rocking platform. For ATP experiments, 4 mM ATP and 5 mM MgCl_2_ were also added to the lysate prior to the addition of antibodies. The protein A/G bead slurry was then added and incubated with the lysate at 4 °C for 2 h on a rocking platform. The beads were then pelleted down by low-speed centrifugation and washed twice with cold PBS. Immunoprecipitated complexes were eluted from the beads by using 100 μl of 0.1% TFA and 50% acetonitrile solution. For immunoblotting samples were prepared by boiling elution fraction with 2X Laemmeli buffer for 30 min at 60 °C. For the preparation of Nano-LC-MS/MS samples, elution fractions were subsequently subjected to in-solution trypsin (Promega, USA) digestion, according to manufacturer’s instruction. Peptides were eluted with 0.1% TFA and desalted with ZipTip (Merck Millipore, USA) using C18 reverse phase chromatographic column. Purified peptides were then spotted on 384 well LC-MALDI stainless steel plates. Peptide fragmented with 4300 laser intensity, and 2500 shots were accumulated for processing of data. Proteins were identified by global analysis using the protein pilot software.

### Isolation of *Chlamydomonas* flagella

*Chlamydomonas* culture was harvested at the cell density of O.D. 600 ~0.7, and the cell pellet was washed with 10 mM Tris (pH 7.8). Cells were resuspended in cold 10 mM Tris (pH 7.8) with 5% sucrose. Cell suspension pH was lowered to pH 4.5 using 0.5 N acetic acid to detach flagella. The pH of the suspension was again raised to 7.0 by 0.5 N KOH. The flagellar fraction was separated by overlaying 15 ml of a sample on 20 ml of 25% sucrose prepared in 10 mM Tris pH 7.8 supplemented with the 0.25X protease inhibitor cocktail (Sigma-Aldrich, USA).

### Immunostaining and confocal imaging of *Chlamydomonas*

Vegetative *Chlamydomonas* cells grown in liquid TAP medium to the cell density of 8 × 10^6^ cells/mL were harvested in microcentrifuge tubes at 3000 rpm for 5 min. The cell pellet was then resuspended in fresh 1XPBS at the cell density of ~10^7^ cells/mL. Aliquots of 120 μL cell suspension were seeded to cover slips pretreated with poly-L-lysine and allowed to settle for 10 min at room temperature. Cells seeded on the coverslip were then treated with freshly prepared 3.7% paraformaldehyde solution in PBS for approximately 4 min. Coverslips were then dipped in ice-cold absolute ethanol solution and incubated at −20 °C for 10 min with brief agitations. After removing ethanol, coverslips were washed with PBS containing 0.5% Triton X-100 (PBST) four times incubated in blocking buffer (1X PBS and 1% BSA) for 1 h at room temperature and then overnight at 4 °C with block buffer containing desired antibody. The fixed cells treated with primary antibody, were then subjected to 5–8 brief washings with PBST followed by four times brief washings with 1X PBS and incubated with Alexa Fluor conjugated secondary antibody (Invitrogen, USA) for 2 h at room temperature. After washing with PBST followed by PBS, coverslips were mounted in the acid washed glass slides using SlowFade^®^ Gold antifade reagent (Molecular Probes, Invitrogen, USA) in the inverted manner. Cells were visualized and imaged with Leica TCS SP5 confocal microscope using 63X oil lens (1.4 numerical aperture, oil immersion objective). Images from Z-series were captured and summed to produce the final image with eyespot and flagellar localization of a protein. The maxima of all the images from Z-series were colored with red for Alexa 546 and green for Alexa 488, merged and adjusted for brightness in Leica processing software (Leica, Germany) or ImageJ software (NIH, USA), and cropped and sized in Photoshop (Adobe Systems Inc.).

### GST pulldown assay

A nucleotide sequence corresponding to full-length IFT20 was cloned in frame with glutathione S-transferase (GST) in a pGEX-4T-1 vector. IFT20-GST fusion protein was produced in *E. coli* BL21 (DE3λ) cells by induction with 0.1 mM IPTG at 16 °C for 48 h. For negative control, GST protein was affinity purified simultaneously under similar conditions using glutathione sepharose 4B (HiTrap affinity columns, GE Healthcare). Total cell lysate of *Chlamydomonas* (CrTCL) was prepared by resuspending 1 g (wet weight) of freshly harvested *Chlamydomonas* culture pellet in 10 ml of cold phosphate buffer saline supplemented with a cocktail of protease inhibitor. The lysate was slightly sonicated and subsequently clarified by centrifugation at 13,300 rpm at 4 °C for 10 min. CrTCL was further pre-cleared by incubating it for 30 min with Glutathione Sepharose beads and 20 μg of GST protein immobilized with 20 μl GST beads, at 4 °C. For the pull down assay, 1 ml of precleared lysate was incubated with IFT20-GST protein and recombinant GST, separately at 4 °C on the rocking platform for 3 h followed by incubation with GST beads for further 1 h. GST beads along with co-precipitated complex were separated by low-speed centrifugation at 3000 rpm for 5 min. Beads were washed three times with cold PBS. Co-precipitated complex with GST beads was resuspended in 2X Laemmeli buffer by incubating at 60 °C for 30 min. Proteins were resolved on SDS-PAGE and analyzed by immunoblotting.

### Proximity Ligation Assay (PLA)

PLA was performed using Duolink II (Sigma, USA) rabbit/goat kit according to manufacturer’s protocol with few modifications. After blocking, incubation with primary antibodies was carried out overnight at 4 °C. PLA plus and minus probes (containing the secondary antibodies conjugated to oligonucleotides) were added and incubated at room temperature for 2 h. Ligase was added, and the ligation reaction was set at room temperature for 2 h. Followed by incubation with the amplification-polymerase solution at room temperature for 2 h, washings with the provided buffers was carried out. Red fluorescence signals were observed by the confocal microscope and visualized with excitation at 543 nm (emission, 585–615 nm).

### Phototaxis assay

Phototactic behavior of the *Chlamydomonas* wild type and mutants were analyzed in petri dishes. All the petridishes with *Chlamydomonas* cells uniformly present throughout the plate were illuminated from one side and the cells were observed for the ability to phototax by observing their accumulation on the other side^66^. Images were captured and compared before light exposure and after 1 h of light exposure. The temperature sensitive IFT mutants were grown at permissive temperature to maintain the flagellated cells.

## Additional Information

**How to cite this article**: Awasthi, M. *et al*. The trafficking of bacterial type rhodopsins into the *Chlamydomonas* eyespot and flagella is IFT mediated. *Sci. Rep.* **6**, 34646; doi: 10.1038/srep34646 (2016).

## Supplementary Material

Supplementary Information
