# Supplementary material for: The trafficking of bacterial type rhodopsins into the Chlamydomonas eyespot and flagella is IFT mediated
Source: Sci Rep. 2016 Oct 3;6:34646. doi: 10.1038/srep34646 (PMC5046144; doi:10.1038/srep34646)
Supplement: Supplementary Information [file srep34646-s1.doc]

# **The trafficking of bacterial type rhodopsins into the *Chlamydomonas* eyespot and flagella is IFT mediated**

Mayanka Awasthi^1†^, Peeyush Ranjan^1†^, Komal Sharma^1^, Sindhu Kandoth Veetil^2^ and Suneel Kateriya^2*^

^1^Department of Biochemistry, University of Delhi South Campus, Benito Juarez Road, New Delhi, India-110021

^2^School of Biotechnology, Jawaharlal Nehru University, New Delhi, India-110067

^†^These authors contributed equally to this work.

^*^Address correspondence to: School of Biotechnology, Jawaharlal Nehru University, New Delhi, India-110067, Email: [skateriya@jnu.ac.in](mailto:skateriya@jnu.ac.in)

**Table 1.** Diverse green algal species possessing modular archaeal type rhodopsin proteins. Abbreviations corresponding to the name of different algal species that are used in this study are mentioned.

| Abbreviations | Organism Name |
| --- | --- |
| ApRh | *Asterochloris sp.* |
| ApRav | *Asterochloris sp.* |
| MpRh | *Micromonas pusilla* |
| BgRh | *Bigelowiella natans* |
| GtRSPEC | *Guillardia theta* |
| GtRh | *Guillardia theta* |
| OtRh | *Ostreococcus tauri* |
| OlRh | *Ostreococcus lucimarinus* |
| Vop | *Volvox carteri* |
| Cop | *Chlamydomonas reinhardtii* |

**Table 2. Details of guanylate cyclase (GC), SMC, and DnaJ proteins that were identified in ChR1-IP-nano-LC-MS/MS.**

| **Name** | **Sequences**  **Yellow region: Peptide identified in ChR1-IP-nano-LC-MS/MS;**  **Blue: antigenic peptide region used for generation of specific antibodies.** |
| --- | --- |
| Guanylate cyclase [*Chlamydomonas reinhardtii*] | >Cre13.g591600.t1.1 MTQLYDMLTPVNTGCDGQLCPESLQRALSELQQEKNRLAEAAKVEQQRLHEELAAARAAKQELEGRVNELAKQLAAAGSAEDDVYDTCRLPVAPLDLSRLALEPDKAAALEARLQAQLQEQQLRPGPGGGAAGGAGEVLDDTVYLPASLDRLQEGPRGLRSVTVDSVRRGAEAEGLRVTLSVLDRVGETDPRATYGAWYRRMSYVSPMYCQHIDAPDEAAAIRTMNVTLANNVVVRSAYTGYLKLLLVGGEPSPAVMRPLVYHSDRRSELVMLWMFPVLVYGTVSELGGEDVAPGDDRMCRRIGLLAFTDPATSAARQFEGMMRDHMGLKYTPSPITMVDEDGVIVTQNPSSACSIGTHGYEARLAGSTRFNYLTQLFASEPGLEADMRRVTAAGGTWSRRLRISDSPILRKWMELEEDEERWHEVQISKLRDPLHLTSSFIIAETDVTATVLAQHEVLRLQRHQQALLKQILPQQVIDVMLSEDRDDDGSGGEQGPRRVAKSMSRRRLGSGVLSRSPIGLGRSARRGRSLGADNVSPRVVAAASGAATVAALAVAAAAGLDGGDIGASGGGGADSCGISSTCSTPRASTRAADSPVPAPQLARAGGSALRRNAAAAFAAAATAAAAAVTANATNTTIATASGASVSSSRASSWHRSCPSSRLSASNLRTEVVAALNASTAAAAAASAAGSAGAGAAPDADCDGGTDDGGCGGDCSPAAAGADAGAGQGSGRHMFGSCADRTMMILMPDGTTHTLDLMDETDGSDSAIQLSRSDVMSLATWHEDVTILFADIKGFTTMSQQLHPARVMLFLDTLYNAFDLLLDECGCYKVETIGDCYMVAGGLFATPDPDGSGEMQLGGYDPDHALKVLRFAIQMADIASRLRTPFGEPVQMRIGIHSGACMSGVVGRRMPRFCLFGDTINTASRMESTGAPGAIHVSEATQRLLPTIKWQPRGEGIEVKGKGLMQTYWWGGDTTNVCFKARDRVIDSVVNRGGRRSATGTGSGEAVDGGGGAGGSGAEAPALTACAEGREEGQQEPAERANGGRLEPLPAAKQAQLEMAEACDTI |
| Structural maintenance of chromosomes protein 3 [*Chlamydomonas reinhardtii*] | >Cre10.g445650.t1.2 MHIKQVLIEGFKSYKDQTSTDEFDPKINVVVGANGSGKSNFFHAIRFVLNDAFINMRGEERLQLLHEGAGHRVSSAWVEVVFDNTDGRFPIDRSEVRLRRTINAKKDDYTLDKKHINKSEVSSLLESAGFSKSNPYYIVQQGKITAMAAMSDAQRMELLKEIGGTRVYEERRKESLRVMQETESRKQQILSMLSEIEDKLRELDAERAELMEYQDLDRRRRCLQYTLFDKELAKATADAARLEREAAQLRETVGSASTDQERTMAEGKELERQVKALEAEYAVAQGQARALQARRQELVAQRSRQEVDIEELERRVRRAESREQSSRREMAALQKDLEAEQAKLKQLQQAAATAEADWSDLQSRIKEADSRLAALYRKQGSSSYRSRDERDAELKKQLVAYEHKLATKKQSRERTQEEYRAHNEQLMELSQTIGDLDAEVRSLEGRVLDSDKAHAEAHAARVKLLDDRKAKQREEEFAEQALKTAEAEMRAAQSAYDKCMPNDVRKGIQGLDALRQRYGVDMSGVHGAVIEHIRIADLFYVAVDTIAGNHLFDVLVENEEVAGRLIRGLHQNNLGRATFVPLNRVGDMPEPPPPTEWGQDVVSLYRKISTDPRFKPAMRDLFGQALLCKDKDVATEVCRSNDKFDCVTLDGEKFGRRGNISGGFAPTNRARLAVYDNLMKAREQVVAADRKAKDVAAEVARLHAVVEAAASQQENLDMERGKLRNVMRDKKQDLKRIREEEAELRSRVDGAERTLTAYESEIAHIEHEVSGLKRELASDMSSTLTAAERNEVKSLNLAKTRMSDQLRGLSQKRDEAQAAVQACEAHITGVLKRREASIQEALSTDDAANDKAALSLRQADLEALRRSLDEAKAEAGRSERRAEELRARLDELQRQRDALRDEAGKREAAVADSAKALEGLDHKREVAAAKAAENERKIRELGSLPQEAFDKPYRDRSIKDLMRALEEVNAGLQRFAGVNRKALDQYVDFSNQREELGSRLKEQQASDSKIRELITALDMRKDEAIERTFKGVAKNFREVFADLVPGGTGELVMIRAAGRAAAADGEDDEGGGTGPSGGSEKYSGVKVKVRFAGAGEAVSMRALSGGQKTLVALALIFAIQRCDPAPFYLFDEIDAALDPQYRTTVAAMLRRQAHDATNPAQFIVTTFHPQIVSEADRLFGVAHTNRISRVYAIQREDALQFLQAAEEHGQQGDGTSHGAGASAAVTKQQGKRARGAAGGASAAAGARKRQRPAARRKEEDEENDNEGSADEEAGGDDDED |
| DnaJ-like protein [*Chlamydomonas reinhardtii*] | >Cre12.g560400.t1.1 MGRSRSRSRERSKRSHHSRSRSRDRDRSERDRRDRRRSRSRDRSERDRRRSRSRSRDRDRSRRSRSRSRDRRDRDRDRDRDRDRAPREGRERSAGGSQPPAAAEANASAGTGASAGASGGAEADRLARLAAWKAKMGGGGAIGAGGAGGAAAAASPAPAAAGPGGSSKVWMPWEDPELVGKGAAAVAAAPAAVAAAPAPQAAAPAAAAAAAGRRPGGGGGGFGLDDEEEEEADRAAKEAEMRAAAIREMMMKDRNYVPPTIKAKKLKDAEHDAELAARAAADDEDPLDAFMAAEVLPEVKQRQAQEEARRQEERRKMAEQLASGKALPSLKLLEELSDDDDPDANPDLEIQIPANKVKLVIGPGGEKIKEIQKKSKARVQVKKDDKELNKGFGEGLKADMEAAMANASGEKKLKTILLFGDEKSVEMAERMIMEAIENKEQKQRNREKEYERKREAKRRERMLYHLRHAKHYELLEVALGASKLDVKKAYRRLAMQWHPDKHPDNQEEAKAKFQEIQKAYEALMSTSEDDIIEQLPDRAGAGAVPEGGVVVPPPAPA |

**Table 3.** Details of different antibodies used in this study. Each row from left to right denotes the name of antibodies used in this study along with the organism in which antibodies were generated and their origin. All the antibodies, which were not commercially available, were generated with the help of Merck-Bangalore Genei, India.

| Name of Antibodies | Host animal | Antigenic region |
| --- | --- | --- |
| ChR1-Ct | Rabbit | ChR1-C-terminal recombinant protein |
| ChR2-Ct | Rabbit | ChR2-C-terminal recombinant protein |
| ChR1-peptide | Rabbit | 10 aa residues peptide of ChR1 |
| ChR2-peptide | Mouse | 10 aa residues peptide of ChR2 |
| Cop8Ex32 | Rabbit | Recombinant protein corresponding to Cop8 exon 32 and 33 |
| Cop8-peptide | Rabbit | 10 aa residues peptide of Cop8 |
| Kinesin-2 | Goat | 15 aa residues peptide of *C. reinhardtii* Kinesin-2 |
| Khp1 | Goat | 15 aa residues peptide of *C. reinhardtii* KHP1 |
| LC8 | Goat | 15 aa residues peptide of *C. reinhardtii* LC8 |
| cPhot | Goat | Recombinant protein corresponding to LOV1 domain of phototropin |
| IFT139 | Goat | 12 aa residues peptide of *C. reinhardtii* IFT139 |
| GC | Goat | 12 aa residues peptide of *C. reinhardtii* Guanylate Cyclase |
| DNAJ | Goat | 12 aa residues peptide of *C. reinhardtii* DNAJ |
| SMC | Goat | 12 aa residues peptide of *C. reinhardtii* SMC |
| FITC | Rabbit | Commercially available from Invitrogen (Molecular probes), USA |
| Alexa 488 | Mouse | Commercially available from Invitrogen (Molecular probes), USA |
| Alexa546 | Rabbit | Commercially available from Invitrogen (Molecular probes), USA |
| Alexa647 | Mouse | Commercially available from Invitrogen (Molecular probes), USA |
| α-Tubulin | Mouse | Commercially available from Sigma Aldrich, USA |
| GST | Mouse | Commercially available from Bio-Rad, USA |
| Penta-His | Mouse | Commercially available from Quiagen, Netherlands |

**Supplementary figures**

**Identification of novel algal rhodopsins**

Chlamydomonas possesses seven different rhodopsins and the genome database search provided a novel modular bacterial rhodopsin in this alga. Furthermore, a comparative analysis of similar modular bacterial rhodopsins from different organisms (see Supplementary Table 1) is presented in Supplementary Fig. 1a-i. In brief, novel modular rhodopsins are associated with domains like Rave, Spectrin, VGKC (Voltage-gated K+ Channel), cNMP (Cyclic nucleotide-binding domain). The newly identified rhodopsin used in this study along with ChR1 is Chlamyopsin8 / Cop8 from *C. reinhardtii*.

**
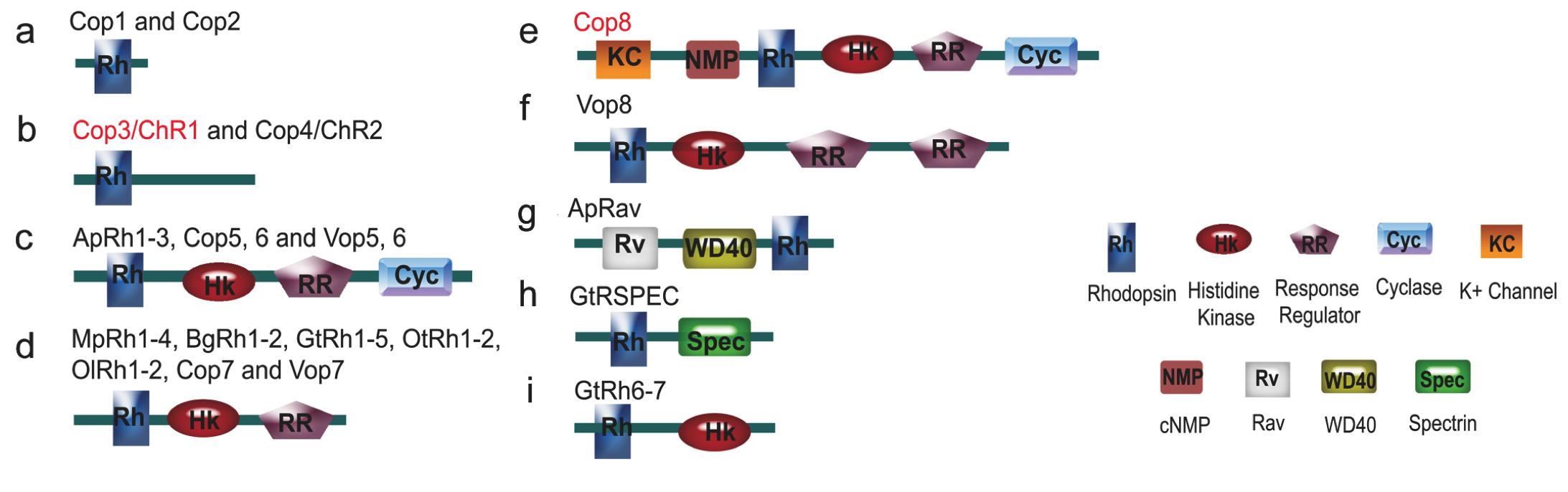
**

**Figure 1:** Schematic representation of the conserved domains of different algal rhodopsins. (a) Cop1 and Cop2 are single domain rhodopsin proteins. (b) Cop3 (ChR1) and Cop4 (ChR2) possess N-terminal rhodopsin (Rh) domain and long C-terminus extension. (c) ApRh1-3, Cop5, 6 and Vop5, 6 proteins from N to C terminus possess Rh, histidine kinase (Hk), response regulator (RR), and cyclase (Cyc) domains. (d) MpRh1-4, BgRh1-2, GtRh1-5, OtRh1-2, OlRh1-2, Cop7 and Vop7 contain Rh, Hk and RR domains. (e). Cop8 is a multidomain protein with potassium channel (KC), cNMP domains at N-terminal of Rh and Hk, RR, Cyc at C-terminal of Rh domain. (f) Vop8 possesses Rh, Hk and two RR domains. (g) ApRav contains N-terminal Rave (Rv), WD40 and Rh at its C-terminus, avii. GtRSPEC contains N-terminal Rh and C-terminal spectrin (Spec) domain. (h) GtRh6-7 possesses N and C-terminal Rh and Hk domains, respectively. Names of diverse algae with respective proteins are listed in Table 1.

**Immunolocalization of Cop8 in *Chlamydomonas* cells.**

Specific antibody generated against Cop8; anti-Cop8-peptide and anti-Cop8Ex32 (Supplementary Fig. 2a), was checked using affinity-purified proteins corresponding to the antigenic regions, see Supplementary Fig. 2b. Cop8 was localized in the flagella and eyespot of the *Chlamydomonas* (Supplementary Fig. 2c). Immunoblotting with the total protein sample of isolated flagella and cell body fractions of *Chlamydomonas* confirmed the presence of Cop8 both in the flagella and cell body of this organism. Both the antibodies identified Cop8 as a protein of approximate molecular weight of 302 kD in *C. reinhardtii* total cell lysate or CrTCL.

**
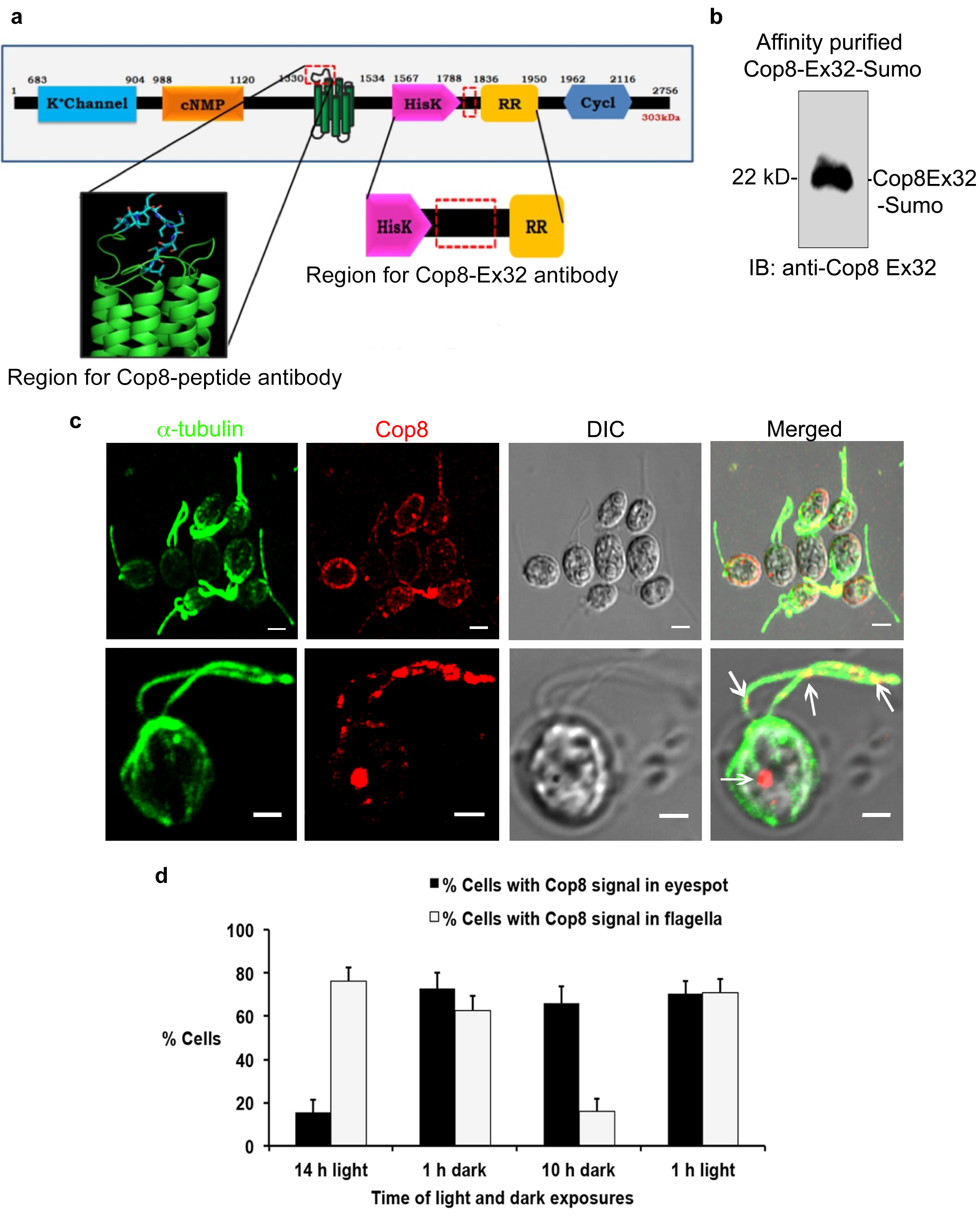
**

**Figure 2** Cop8 localizes in the eyespot and flagella of *Chlamydomonas reinhardtii*. (a) Schematic of the antigenic regions used for specificity of Cop8-peptide (blue) and Cop8Ex32 (red) antibodies. (b) A protein band (22 kD) equivalent to approximate molecular weight of affinity purified recombinant Cop8Ex32-Sumo was detected in IB with Cop8Ex32 antibody. (c) Cellular localization of α-tubulin (green) with monoclonal anti-α-tubulin antibody and Cop8 (red) with an anti-Cop8Ex32 antibody. Cop8 localizes in the eyespot as well as flagella of the *C. reinhardtii* cell. Differential interference contrast (DIC) microscopy image indicates eyespot as a black spot in each cell. Green and red channels merged with DIC are also presented. Scale bar: 2 µm. Magnified image of single *C. reinhardtii* cell (lower panel). Green and red channels represent α-tubulin and Cop8, respectively. Flagellar localization of Cop8 is indicated by arrowhead in merged image. (d) Bar graph represents the effect of different light-dark conditions in the trafficking of Cop8 as quantified from 5 independent experiments. Error bars represent standard deviation (s.d.).


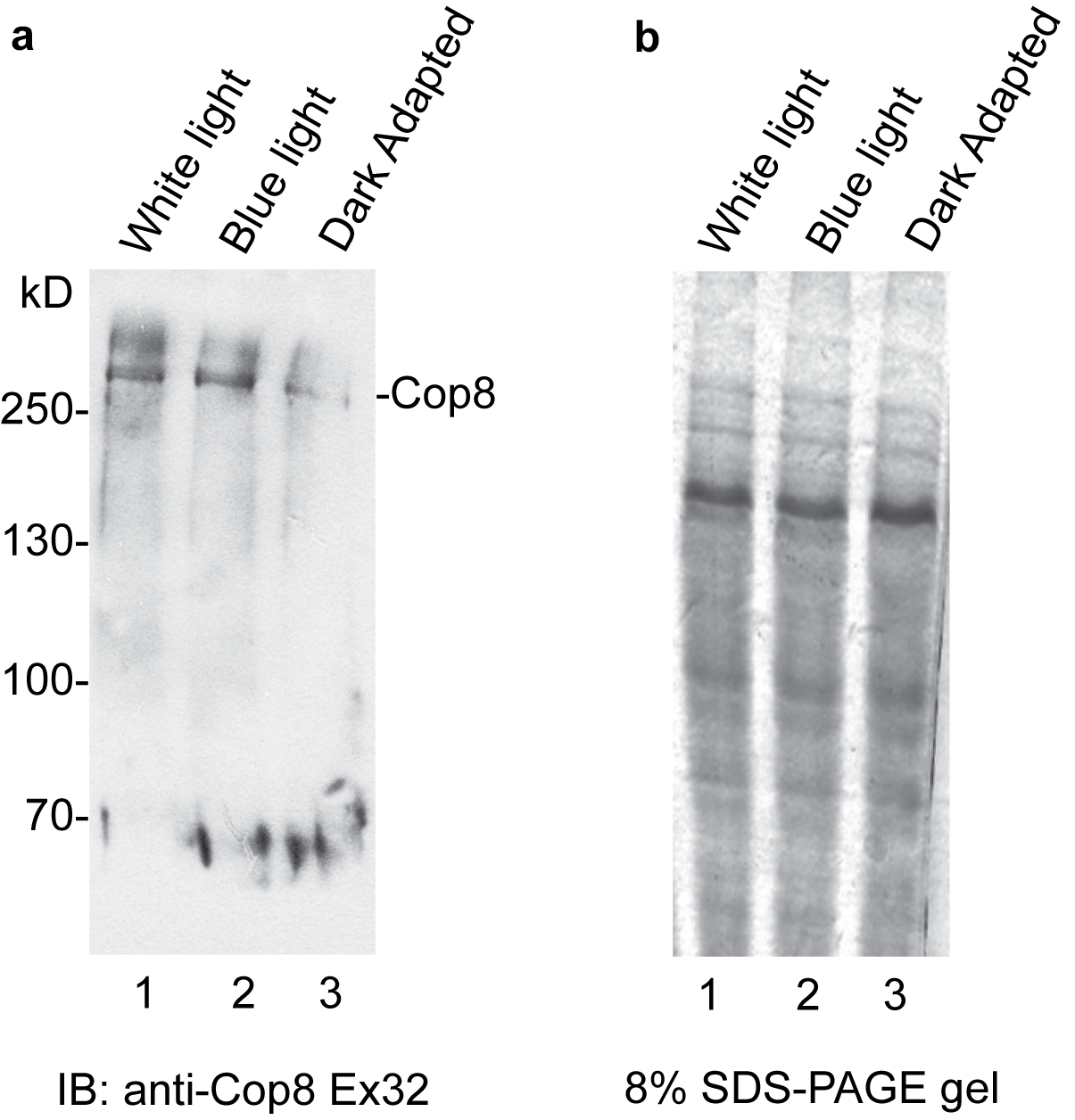


**Figure 3** Immunoblotting of Cop8 in isolated flagellar fractions of *C. reinhardtii*. (a) Cellular detection of Cop8 using Cop8-Ex32 antibody in the flagella of the cells grown in white light, blue light and in dark. (b) SDS-PAGE profile of the flagellar fractions, serving as loading control.


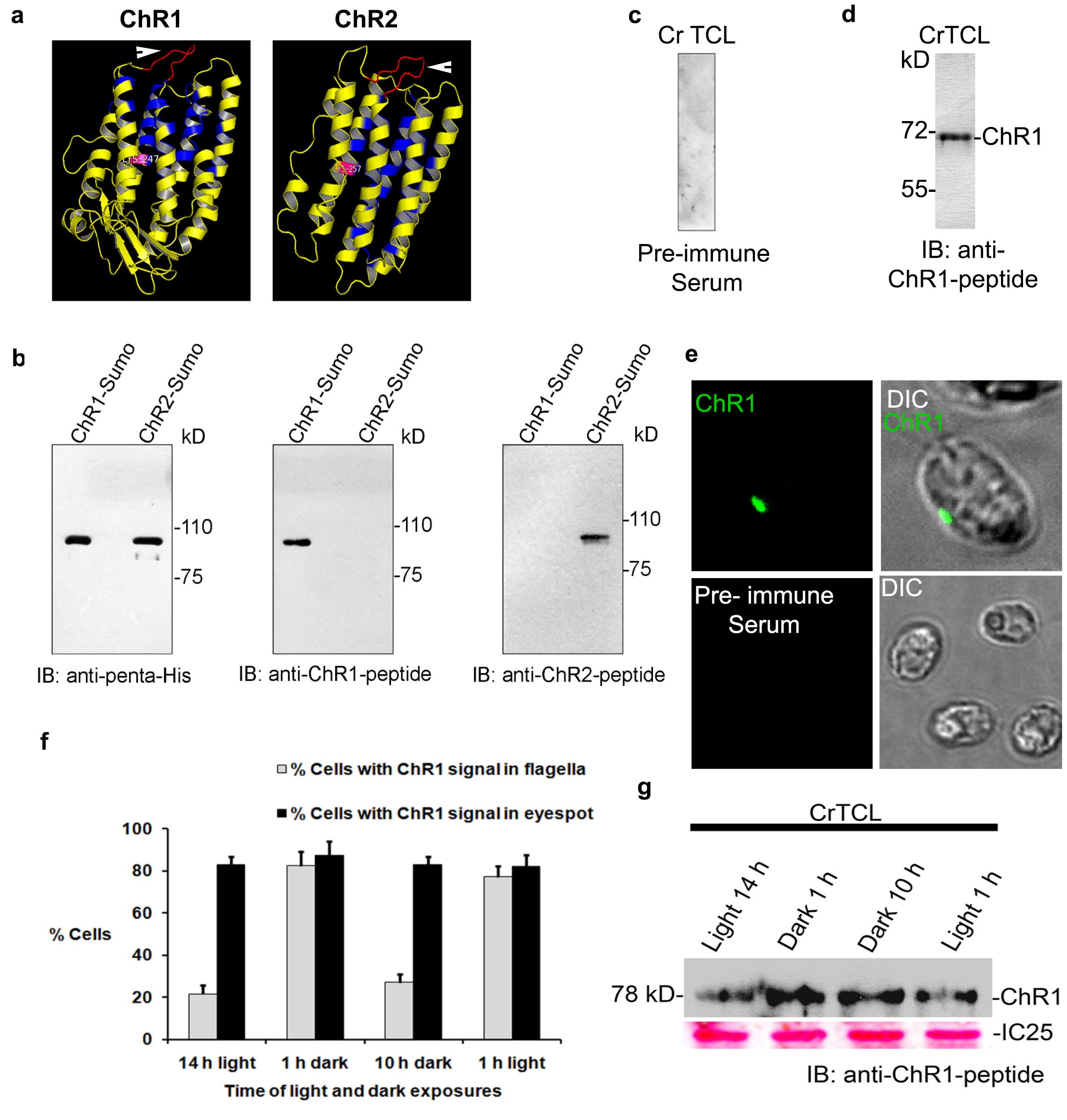


**Figure 4:** (a) Cartoon representation of predicted tertiary structures of ChR1 and ChR2 with the antigenic regions used for generating ChR1-peptide and ChR2-peptide antibodies (red). (b) Immunospecificity of ChR1 and ChR2-peptide antibodies. From left to right represent immunodetection of affinity purified ChR1-Sumo and ChR2-Sumo using penta-His antibody, which recognized both proteins. Immunoblotting of the same membrane after stripping with ChR1-peptide antibody recognized ChR1 and re-probing with ant- ChR2 recognized ChR2 protein. (c) No protein band was detected by immunoblotting of CrTCL using rabbit pre-immune serum. (d) Single protein band corresponding to ChR1 was detected in CrTCL using anti-ChR1 peptide antibody. (e) Cellular localization of ChR1 using the ChR1-peptide antibody. ChR1 (green) merged with DIC showed localization of ChR1 in the eyespot. Cellular localization in *C. reinhardtii* using the preimmune serum and its DIC image showed no specific fluorescence from any part of the cell (lower panel). (f) The bar graph represented the effect of different light-dark conditions in the trafficking of Cop8 as quantified from 5 independent experiments. Error bars represent standard deviation (s.d.). (g) Cellular detection of ChR1 at different time points of the light/dark cycle using the ChR1-peptide antibody. The time of sampling is indicated above each lane. Expression of ChR1 was observed to be high during the dark period.


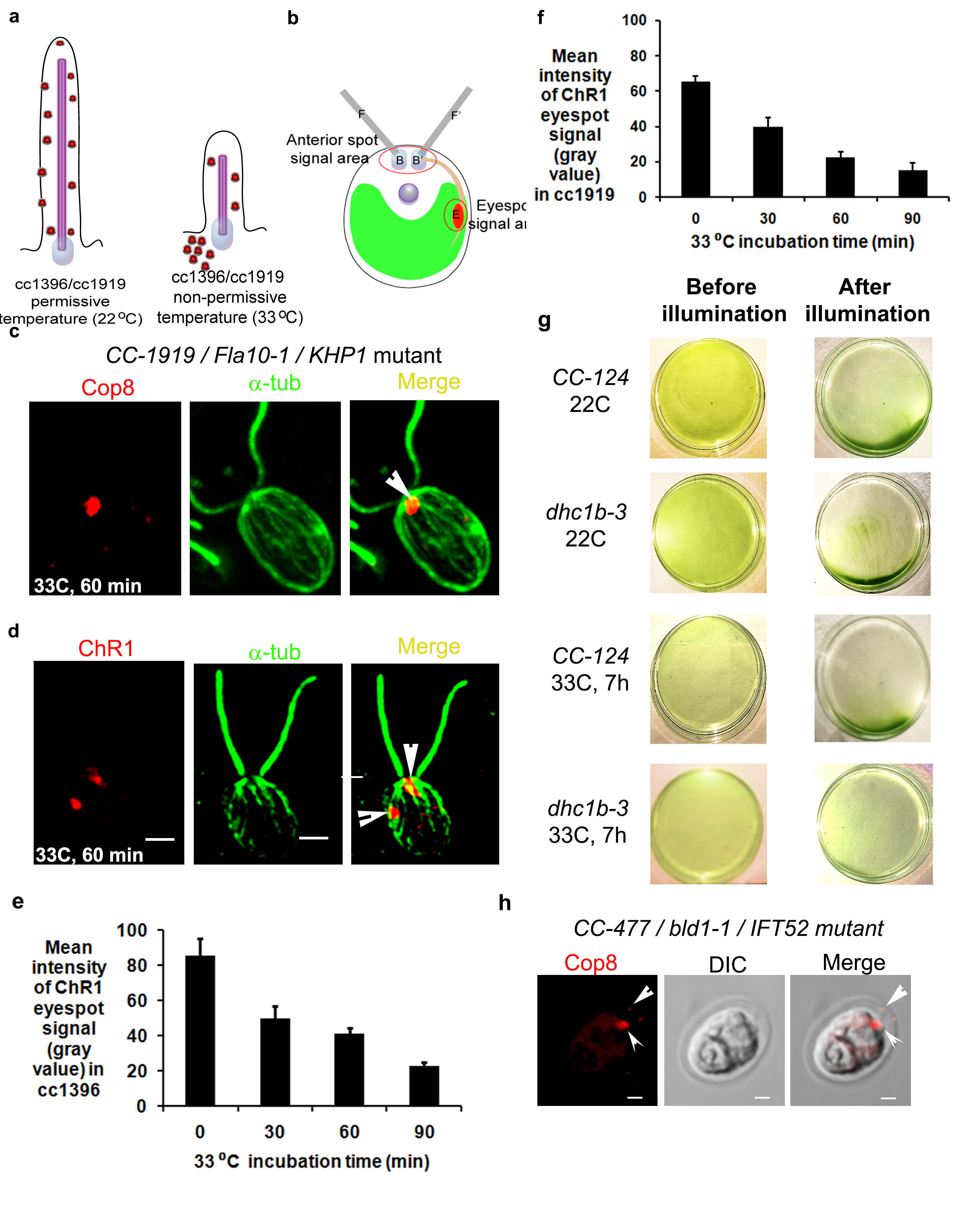


**Figure 5** Defects in IFT motor mutants affect the flagellar trafficking of Cop8 and ChR1. (a) Schematic representation of the trafficking of cargo proteins in the flagella of temperature sensitive IFT motor mutants of *Chlamydomonas* (*CC-1919/fla10-1* and *CC-1396/fla8*), under permissive (22^0^C) and non-permissive (33^0^C) temperatures. (b) Schematic representation of the selected anterior spot and eyespot region (marked under red circle) for calculation of mean signal intensity (gray value). E, B, and F represent eyespot, basal bodies, and flagella respectively. Chloroplast and nucleus of the cell are shown in green and blue respectively. (c and d) Cellular localization of Cop8 and ChR1 in fla10-1 cells grown at non-permissive temperature for 1 h. Accumulation of protein near the basal bodies is shown by arrowhead. (e and f) Quantification of ChR1 signal at eyespot in *CC-1396/fla8* and *CC-1919/fla10-1* at different time points of incubation under the non-permissive temperature. Signals were quantified from 5 independent experiments. Error bars represent standard deviation (s.d.). (g) Comparative phototaxis analysis of *Chlamydomonas* wild-type (WT) *CC-124* and *dhc1b-3* mutant. (h) Immunolocalization of Cop8 in *bld1-1* mutant strain defective in IFT52. Cop8 was accumulated in basal bodies and peribasal region. Each panel represents *Chlamydomonas* strains, WT and mutant with strain name and growth temperature conditions, named at the left of each panel. Images were captured right before illumination and, i.e., at 0 min (before light) and after 30 min of illumination (after light) of directional light exposure, respectively.


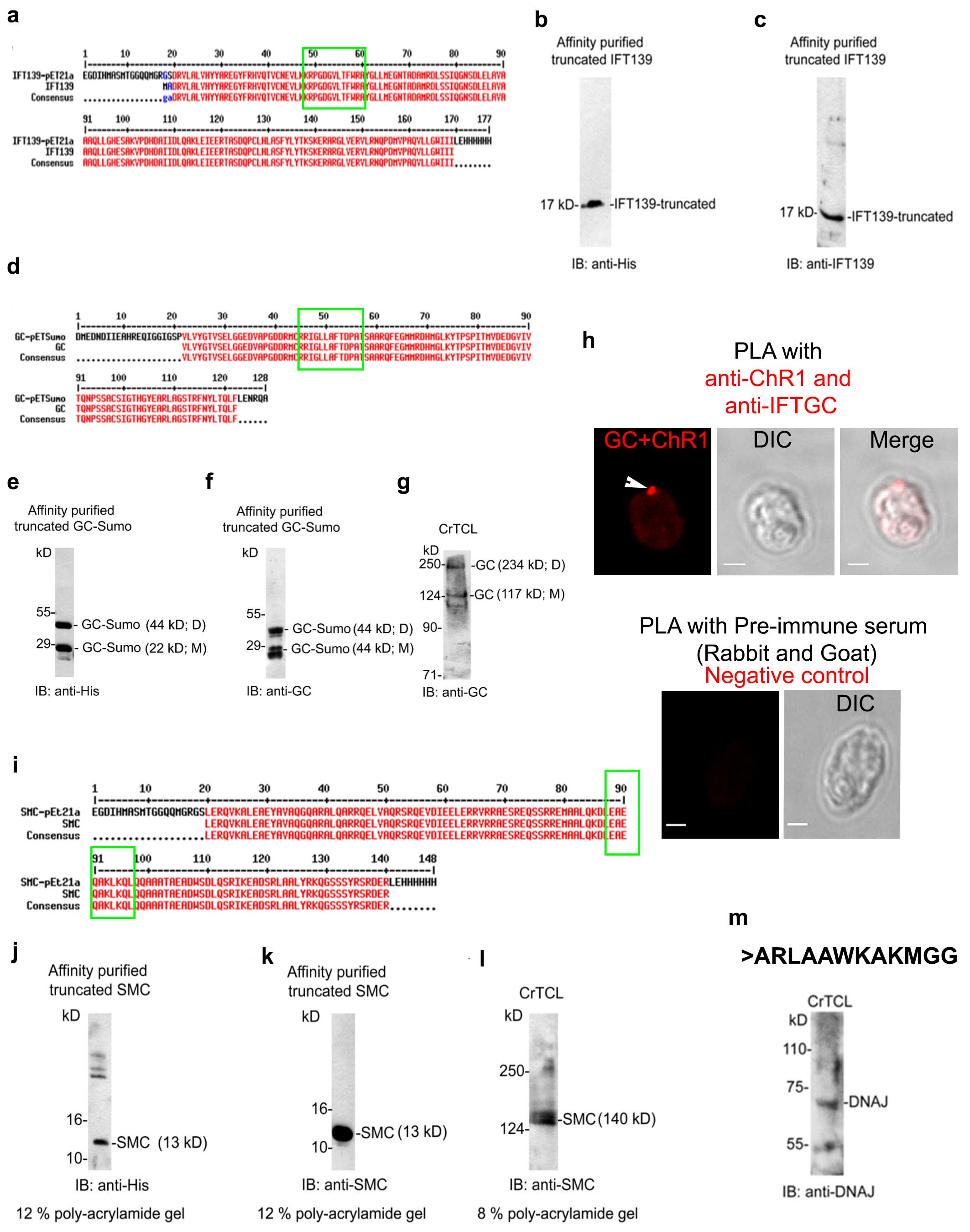


**Figure 6**: Specificity of antibodies against IFT139, GC, SMC and DnaJ. (a) IFT139 protein sequence with the antigenic peptide region (green). (b and c) anti-His and anti-IFT139 recognized same band corresponding to recombinant IFT139 fragment by immunoblotting. (d) Guanylate Cyclase (GC) protein sequence with the antigenic peptide region (green). (e and f) anti-His and anti-IFT139 recognized similar bands corresponding to size of monomer and dimer of recombinant GC fragment by immunoblotting. (g) Cellular detection of GC in CrTCL by immunoblotting with anti-GC also detected bands equivalent to the size of monomer and dimer. (h) PLA with anti-ChR1 and anti-GC together with DIC and merged image (upper panel). Arrowhead indicates the presence of red spot near basal bodies. Negative control includes the PLA with pre-immune serum together with DIC image, which showed no fluorescence. Bar=2 μm. (i) SMC protein sequence with the antigenic peptide region (green). (j and k) anti-His and anti-IFT139 recognized similar protein band corresponding to size of SMC fragment by immunoblotting. (l) Cellular detection of SMC from CrTCL by immunoblotting with anti-SMC. (m) Antigenic peptide sequence of DnaJ, anti-DnaJ identified protein band corresponding to size of DnaJ (62 kD) from CrTCL by immunoblotting.

**
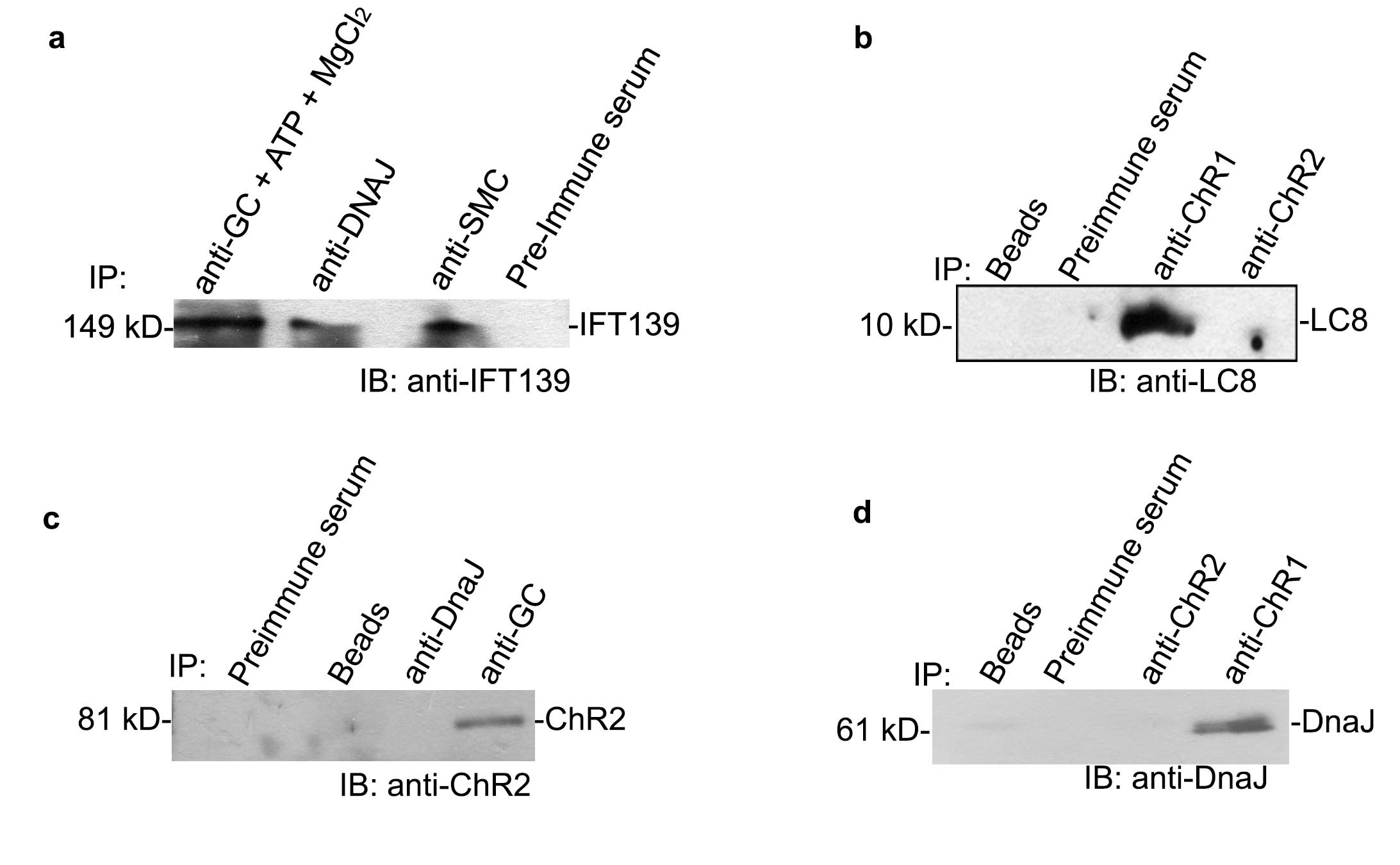
**

**Figure 7:** IFT139 associates with GC, SMC and DnaJ. (a) Immunodetection of IFT139 in IP eluent fractions of GC, DnaJ and SMC. (b) Immunodetection of LC8 in ChR1 and ChR2 IP fractions. Immunobloting with anti-LC8 suggested that the LC8 interacts specifically with ChR1 (c) Immunodetection of ChR2 in DnaJ and GC immunoprecipitation elution fractions. ChR2 associates with GC but not with DnAJ (d) Immunodetection of DnaJ in immunoprecipitation elution fractions of ChR1 and ChR2. DnaJ was identified in ChR1-IP but not in ChR2-IP fractions. Antibodies corresponding to the IP-elution fractions present in each lane are as mentioned above each lane.
